# Genome-Wide Differentially Methylated Region Analysis to Reveal Epigenetic Differences of Articular Cartilage in Kashin–Beck Disease and Osteoarthritis

**DOI:** 10.3389/fcell.2021.636291

**Published:** 2021-03-01

**Authors:** Yue Fan, Dalong Gao, Yingang Zhang, Jiaqiang Zhu, Feng Zhang, Lu Wang, Yan Wen, Xiong Guo, Shiquan Sun

**Affiliations:** ^1^School of Public Health, Health Science Center of Xi'an Jiaotong University, Xi'an, China; ^2^Key Laboratory of Trace Elements and Endemic Diseases of National Health Commission and Collaborative Innovation Center of Endemic Diseases and Health Promotion in Silk Road Region, Xi'an, China; ^3^Department of Orthopaedics, The Second Affiliated Hospital of Xi'an Jiaotong University, Xi'an, China; ^4^Department of Orthopaedics, The Central Hospital of Xianyang, Xianyang, China; ^5^Department of Orthopaedics, The First Affiliated Hospital of Xi'an Jiaotong University, Xi'an, China; ^6^Department of Biostatistics, University of Michigan, Ann Arbor, MI, United States; ^7^Department of Biostatistics, School of Public Health, Cheeloo College of Medicine, Shandong University, Jinan, China

**Keywords:** differentially methylated region, Kashin–Beck disease, osteoarthritis, articular cartilage, DNA methylation, cartilage

## Abstract

Kashin–Beck disease (KBD) is a degenerative osteoarticular disorder, and displays the significant differences with osteoarthritis (OA) regarding the etiology and molecular changes in articular cartilage. However, the underlying dysfunctions of molecular mechanisms in KBD and OA remain unclear. Here, we primarily performed the various genome-wide differential methylation analyses to reveal the distinct differentially methylated regions (DMRs) in conjunction with corresponding differentially methylated genes (DMGs), and enriched functional pathways in KBD and OA. We identified a total of 131 DMRs in KBD vs. Control, and 58 DMRs in OA vs. Controls, and the results demonstrate that many interesting DMRs are linked to DMGs, such as *SMOC2* and *HOXD3*, which are all key genes to regulate cartilage/skeletal physiologic and pathologic process, and are further enriched in skeletal system and limb-associated pathways. Our DMR analysis indicates that KBD-associated DMRs has higher proportion than OA-associated DMRs in gene body regions. KBD-associated DMGs were enriched in wounding and coagulation-related functional pathways that may be stimulated by trace elements. The identified molecular features provide novel clues for understanding the pathogenetic and therapeutic studies of both KBD and OA.

## Introduction

Kashin–Beck disease (KBD) is an endemic and chronic osteoarthropathy, widely affects the population in certain areas of Russia, North Korea, and China (Stone, 2009). According to China Health and Family Planning Statistical Yearbook 2016, over 60,000 people are currently affected by KBD, and over 1.1 million people are potentially affected by KBD (Commission, 2016). In contrast, osteoarthritis (OA) is also a chronic and degenerative joint disease, and affects the population in the worldwide, over 250 million people are currently affected by OA (Mora et al., 2018). Although KBD and OA share several common characteristics in manifestation and pathological of articular cartilage, extensive studies have shown the significant differences in the etiology and molecular mechanism (Schepman et al., 2011).

KBD is diagnosed as an environmental-affected disease, and more than 50 potential environmental risk factors have been investigated, including iodine and selenium deficiency (Moreno-Reyes et al., 1998), the fungal mycotoxins (Peng et al., 1992; Yang et al., 2017), and the humic acids in drinking water (Guo et al., 2014), etc. The degenerative changes in KBD first appear in the deep zone (approximately 30–40% of articular cartilage thickness) of the cartilage and starts early in children as young as 2- or 3-year-old, while OA is diagnosed as the age-related disease and frequently compounded by various risk factors and biological age-related changes, including high-impact sports (Saxon et al., 1999), cardiovascular disease (Visman et al., 2019), and diabetes (Piva et al., 2015), etc. The degenerative changes in OA first appear in the superficial (i.e., tangential) zone (approximately 10–20% of articular cartilage thickness), and increases rapidly between the 50- and 75-year olds. Therefore, clarifying the pathogenetic similarities and differences between KBD and OA may provide novel clues for understanding the distinct pathogenesis of the both.

DNA methylation is associated with transcriptional regulation, a major epigenetic factor often involving in gene regulation and cell differentiation during chondrocyte differentiation (Compere and Palmiter, 1981; Miranda-Duarte, 2018). For example, methylcytosine dioxygenase TET1 is an important enzyme in catalyzing cytosine demethylation in chondrocytes (Rice et al., 2020); the KBD-specific differentially methylated site cg25325949, located in the *HAPLN1* gene body, is a key component of cartilage extracellular matrix (Wang et al., 2017), etc. However, most studies of DNA methylation profiling have shown that functional changes occur in particular regions, such as promoters, CpG islands (Jaenisch and Bird, 2003) or CpG island shores, and replication of differentially methylated regions (DMRs) are often more successful than that changes at individual sites (Ventham et al., 2016). In addition, statistically, we found that the statistical power in testing a single CpG site might be too small to detect, especially for small sample size studies (e.g., five cases vs. five controls). In contrast, interrogating the CpG sites within a particular region rather than individual CpG site would be able to improve the sensitivity and specificity for testing epigenetic alterations in tissue differentiation (Yan et al., 2020).

In this paper, we performed a comprehensive differential methylation region analysis on genome-wide DNA methylation data from five KBD patients, five OA patients, and five healthy subjects. We first illustrated that the DNA methylation levels of OA is highly correlated with age, while KBD is not. Next, we performed the paired differentially methylated region analyses to show the distinct epigenetic alternations in KBD and OA. Finally, we annotated the DMRs to the associated differentially methylated genes (DMGs) to examine the exact role of DNA methylation in gene expression, and further performed the enrichment analysis to uncover the distinct functional pathways in KBD and OA.

## Materials and Methods

### Sample Collections

The knee cartilage specimens of five KBD patients, five OA patients, and five healthy controls were collected from the femoral condyles of knee joints undergoing total knee joint arthroplasty, and the normal knee cartilage specimens were collected from subjects undergoing amputation caused by traffic accidents. All study subjects were Chinese Han and matched with age and sex, and with no other bone or joint disease history. All subjects were dissected and rapidly frozen in liquid nitrogen. DNA was then extracted from the cartilage specimens using QIAamp DNA Mini Kit (QIAGEN, Germany) following the standard protocol. Agarose gel electrophoresis was employed to evaluate the quality of extracted DNA.

### Genome-Wide DNA Methylation Profiling

Genome-wide DNA methylation profiling was performed using the Illumina Infinium HumanMethylation450 BeadChip, resulting in 485,577 methylation sites across the whole genome. According to the standard protocol for the EZ DNA Methylation Kit (Zymo Research, USA), 500 ng DNA was used for bisulfite conversion, amplified, hybridized to the HumanMethylation 450 array, stained, and washed. The raw image intensities were scanned using iScan SQ scanner (iScan System, Illumina USA). Raw DNA methylation data were processed using *Minfi* package (version 1.32.0) (Aryee et al., 2014). Specifically, methylation probes with intensities indistinguishable from that of the background (detection *p-value* > 0.01) in more than one sample were excluded. Besides, SNP probes, cross-reactive probes, probes with a bead count <3 in at least 5% of samples and the probes located on the X and Y chromosomes were also removed, resulting in 405,389 total probes, with each probe corresponding to a CpG site. Those 405,389 probes could be further annotated to a total of 19,553 genes.

### DNA Methylation Level Measurements

To perform the methylation data using existing methods (e.g., *limma*), DNA methylation level measurement is a necessary step prior to downstream analysis. There are two commonly used DNA methylation measurement methods, β − *value* and *M* − *value* (Du et al., 2010), to measure the methylation levels from microarray-based Infinium methylation assay. *M* − *value* is a logit-transformed of β − *value*, i.e., $M-value=log\left(\frac{\beta }{1-\beta }\right)\;$. The range of β − *value* is from 0 to 1 and shows the more intuitive biological interpretations, while the range of *M* − *value* is from −∞ to +∞ and shows more statistical stabilities. The previous study utilized the β − *value* to perform the differentially methylated analysis, showing β − *value* severely compressed at the low and high when compared with *M* − *value*, as well as its bimodal distribution was less obvious. Therefore, *M* − *value* may provide more insights into the methylation level difference between case and control across the genome, but it is difficult to visualize such difference using β − *value* measurement (Supplementary Figure 1). Therefore, we used *M* − *value* as the measurements for methylation level for downstream analysis.

### Differentially Methylated Regions

To identify DMRs that were related to KBD and OA patients, we first performed the empirical Bayes moderated *t-test* implemented in *limma* package (version 3.42.2) and controlled the effects of age and sex as covariates. We then performed *ProbeLasso* function (Butcher and Beck, 2015) implemented in the R/Bioconductor package ChAMP (version 2.16.2) (Tian et al., 2017) to detect DMRs. The parameter settings of *ProbeLasso* were set as default, i.e., the lasso radius is equal to 375, the minimum separation (bp) between neighboring DMRs is equal to 1,000, and the minimum DMR size (bp) is equal to 50. For significant DMSs (differential methylated sites), we used a threshold of false-discovery rate (FDR) < 0.05 after correcting by the Benjamini–Hochberg method (Benjamini and Hochberg, 1995), while the DMRs were defined as significant regions if the DMR contained more than one site, and the FDR was <0.05. We defined two criteria to select the significant DMSs: (1) a stringent criterion required both statistical and biological significance (Lacey et al., 2019), i.e., an absolute percent methylation difference (PMD) of >20% in the paired groups; (2) a less-stringent criterion only required statistical significance, i.e., it only depended on statistically significant FDR. We defined that the DMRs were overlapped if the width of the overlapped part exceeded 50% compared with the narrower region. We measured the overlapped conditions between KBD-associated DMG/DMR and OA-associated DMG/DMR using the Jaccard index:

$$\text{Jaccard }\left(\text{A},\text{B}\right)=\frac{\left|\text{A}\cap \text{ B}\right|}{\left|\text{A}\cup \text{B}\right|}$$

where |A| denotes the number of elements in set A.

### Gene Set Enrichment Analysis

The DMGs are defined as the genes that were annotated to the differentially methylated regions with PMD larger than 20%, which had additional biological significance. With the significant DMGs, we then performed gene ontology (GO) and Kyoto Encyclopedia of Genes and Genomes (KEGG) pathway enrichment analysis using enrichGO (a total of 10,271 GO terms) and enrichKEGG (a total of 229 pathways) functions implemented in R package *ClusterProfiler* (version 3.14.3) (Yu et al., 2012). We used the 19,553 genes covered by the microarray as the background genes, and set the minimal and maximal sizes of genes annotated by Ontology term for testing as 10 and 500, respectively. The significant GO terms or KEGGs were selected with the *q*-values <0.05.

## Results

### Clinical Characteristics of the Study Patients

The study involved five KBD patients, five OA patients, and five healthy controls, measured by genome-wide DNA methylation profiling. The average ages of KBD patients, OA patients, and normal individuals were 57.4 ± 7.13 years old, 64.6 ± 5.18 years old, and 51 ± 7.31 years old, respectively. The detailed clinical characteristics of the studied people are summarized in Table 1.

**Table 1:** Basic characteristics of study subjects for genome-wide DNA methylation profiling in this study.

**Trait**	**Age**	**Sex**	**#CpG sites**
KBD	51	Female	405,389
KBD	55	Female	405,389
KBD	69	Male	405,389
KBD	59	Male	405,389
KBD	53	Male	405,389
OA	58	Female	405,389
OA	62	Female	405,389
OA	72	Male	405,389
OA	66	Male	405,389
OA	65	Male	405,389
Normal	47	Female	405,389
Normal	62	Female	405,389
Normal	55	Male	405,389
Normal	46	Male	405,389
Normal	45	Male	405,389

### DNA Methylation Levels Is Highly Correlated With Age in Osteoarthritis but Not in Kashin–Beck Disease

**Figure 1:**
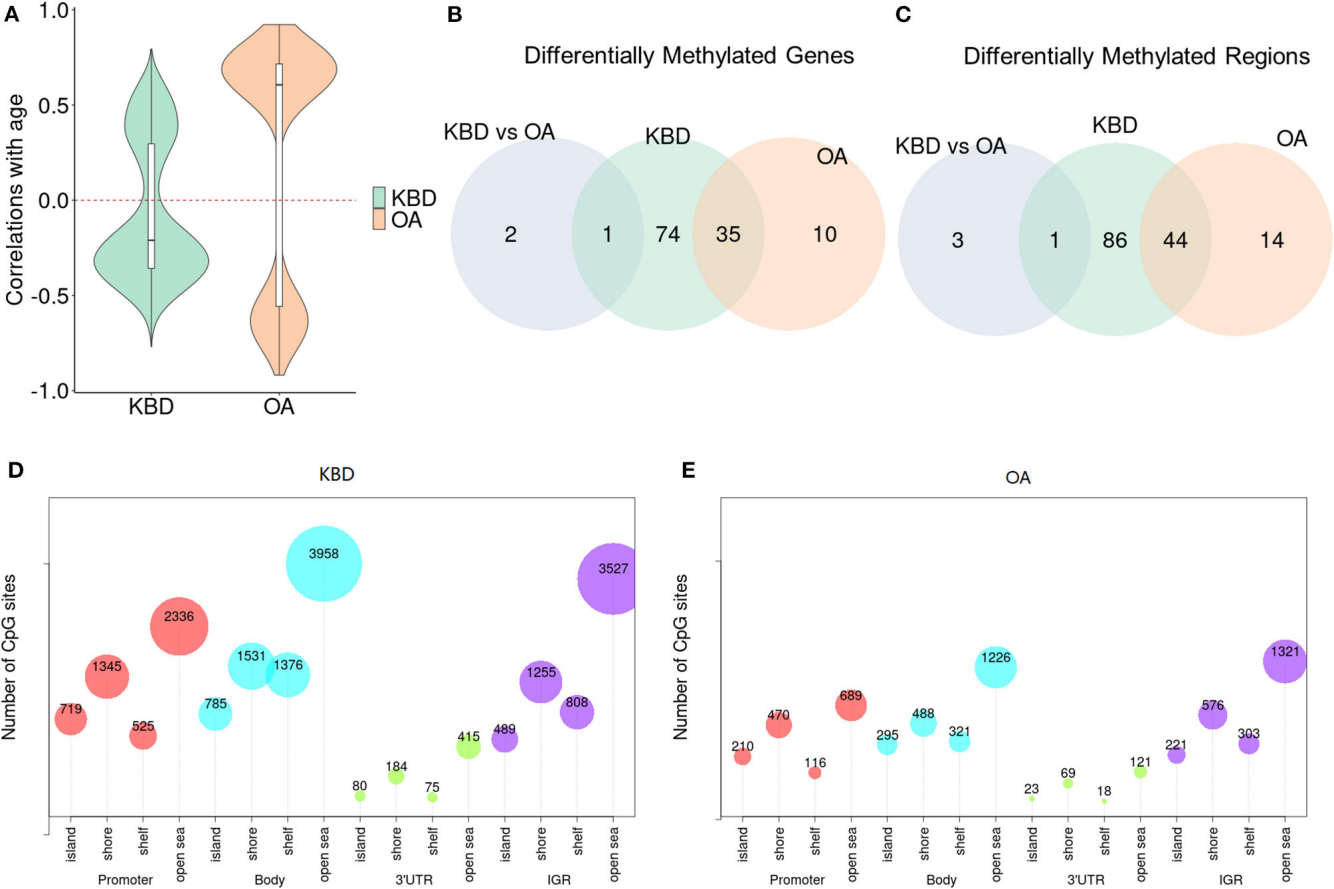
The differences of Kashin–Beck disease (KBD) and osteoarthritis (OA) in differentially methylated regions (DMRs) and differentially methylated genes (DMGs). **(A)** Violin plots show the Pearson correlations between age and the significant DMSs without accounting for age as covariate. OA shows the higher correlations while KBD displays the lower correlations (either positive or negative). **(B)** Venn diagram shows the overlap of the DMRs between KBD vs. Controls, OA vs. Controls with large absolute percent methylation difference (PMD) (i.e., larger than 20%). **(C)** Venn diagram shows the overlap of the corresponding DMRs annotated to DMGs with large PMD (i.e., larger than 20%) between KBD vs. Controls, OA vs. Controls. **(D)** The bubble plot to show CpG sites harbored in the DMRs of KBD vs. Controls. **(E)** The bubble plot to show CpG sites harbored in the OA vs. Controls. PMD, absolute percent methylation difference.

We first examined the relationship between OA/KBD and ages. To do so, we performed a standard linear regression model on the methylation levels as the response variable, and the disease status as the predictor variable, and controlled ages as the covariate to examine whether the number of identified DMSs will reduce or not. As expected, for KBD vs. Controls, when age was excluded in the regression model, 12,666 DMSs were detected; however, if age was included as a covariate, the number of DMSs were reduced to 4,325 (i.e., roughly 65.9% decreased). In contrast, for OA vs. Controls, when age was not included as covariate, 3,499 sites were detected, while only one site was detected if age was included as covariate (i.e., roughly 100% decreased). No DMSs detected in OA vs. Controls was presumably due to OA is an age-related disorder (Anderson and Loeser, 2010). In addition, we performed the Pearson correlation between age and the genome-wide DNA methylation sites. The median absolute correlation was 0.34 (mean value was 0.34) for KBD, while the median absolute correlation was 0.67 (mean value was 0.66) for OA (Figure 1A and Supplementary Figure 2). Note that the age between OA and Control showed significant difference (i.e., 64.6 ± 5.18 vs. 51 ± 7.31; two sample *t*-test *p-value* = 1.1 × 10^−2^). Consequently, if performing the differential methylation analysis did not account for the age, most of the DMSs were detected in OA vs. Controls due to the age discrepancy between OA vs. Controls.

In addition, we extracted the top 10 methylation principal components based on M-values to examine the batch differences among KBD, OA, and Control samples. A heatmap showed that the batch differences among KBD, OA, and Control samples were not significant (Supplementary Figure 3).

### Kashin–Beck Disease Associated Differentially Methylated Regions Has Higher Proportion Than Osteoarthritis-Associated DMRs in Gene Body Regions

Next, to investigate the differences of OA and KBD in DMRs, we performed three groups of DMR analyses, i.e., KBD vs. Controls, OA vs. Controls, and KBD vs. OA. Consequently, 3,009 DMRs (19,408 CpG sites harbored) were identified for KBD vs. Controls with a threshold FDR < 0.05, including 1,012 hypermethylated regions and 1,997 hypomethylated regions; for OA vs. Controls, 1,063 DMRs (6,467 CpG sites harbored) were identified, including 536 hypermethylated regions and 527 hypomethylated regions; and for KBD vs. OA, 399 DMRs (2,939 CpG sites harbored) were identified, including 290 hypermethylated regions and 109 hypomethylated regions. Most of the CpG sites were located at the body region and intergenic region (IGR). Such results were consistent with previous studies (van den Dungen et al., 2017). For example, there were 3,958 total CpG sites in open sea of the body region and 3,527 CpG sites in open sea of the IGR region (Figures 1D,E and Supplementary Figure 4). Methylation that occurred in the gene body regions could affect gene expression by stimulating transcription elongation or alternative splicing (Maor et al., 2015), and generally positively correlated with gene expression (Yang et al., 2014); therefore, it was crucial for gene regulation and genomic stability (Jones, 2012). The overlapping of DMRs between KBD and OA was 719 (Jaccard index = 0.103; Fisher's exact test: *p* − *value* < 2.2 × 10^−16^). The KBD- and OA-specific regions were 2,290 and 344, respectively (Supplementary Figure 5A). We observed that the proportion of hypomethylated regions of KBD vs. Controls (66.4%) located in the gene body regions was higher than the OA vs. Controls (50.4%), presumably implying that the epigenetic factors may repress more gene expression in KBD than OA. Besides, for KBD vs. OA, 49.5% hypomethylated regions were located in gene body regions, while 33.3% hypermethylated regions were located in gene body regions.

In addition, we further detected DMRs using the stringent criterion that required both statistical and biological significance. With this criterion, we identified 131 DMRs (548 CpG sites harbored) in the KBD vs. Controls, including 62 hypermethylated regions and 69 hypomethylated regions (Supplementary Table 1); 58 DMRs (293 CpG sites harbored) in the OA vs. Controls, including 37 hypermethylated regions and 21 hypomethylated regions (Supplementary Table 2), and four DMRs (24 CpG sites harbored) in the KBD vs. OA group, including three hypermethylated regions and one hypomethylated region (Supplementary Table 3). The overlapping of DMRs between KBD and OA was 44 (Jaccard index = 0.303; Fisher's exact test: *p* − *value* < 2.2 × 10^−16^). The KBD- and OA-specific DMRs were 87 and 14, respectively (Figure 1B). We observed that the proportion of KBD-associated DMRs (67.9%) located in the gene body regions is higher than that of OA-associated DMRs (32.8%), presumably implying that the epigenetic factors played a more important role in KBD than OA. We did not further examine the proportion of DMRs of KBD vs. OA since we only identified four DMRs.

### Kashin–Beck Disease and Osteoarthritis-Associated Differentially Methylated Genes Were Enriched in Different Functional Pathways

**Figure 2:**
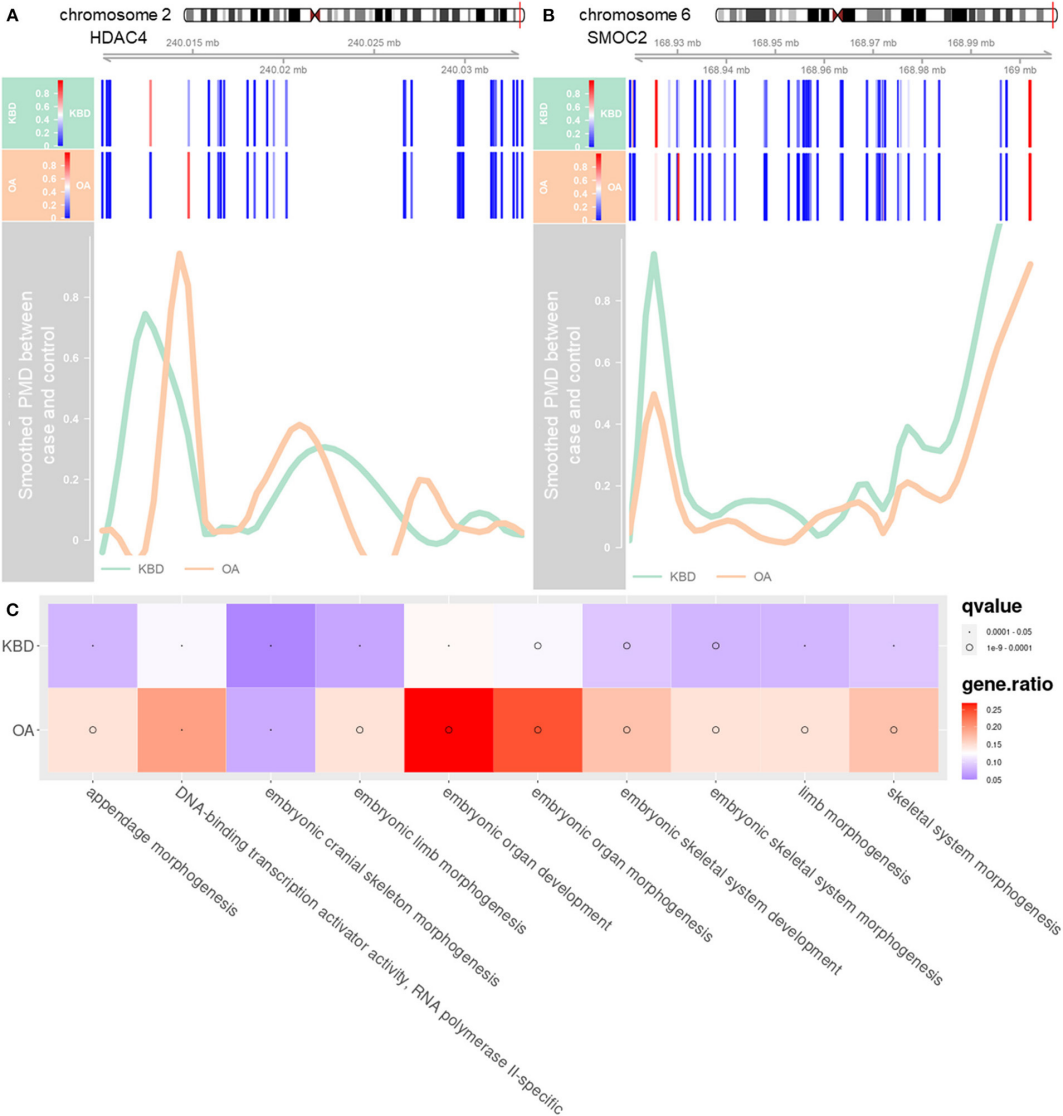
The distribution of methylation levels in DMGs and enriched significant pathways of both KBD and OA. **(A)** DMR annotated to DMG HDAC, which is located in chromosome 2 (position: 240,014,758–240,028,276). **(B)** DMR annotated to DMG SMOC2, which is located in chromosome 6 (position: 168,925,150–169,002,604). The *x*-axis represents the coordinate of chromosome and *y*-axis represents PMD after smoothing. The green line represents the PMD between KBD vs. Controls and the orange line represents the PMD between OA vs. Controls. The blue bars represent the CpG sites. Note that the green line and orange line would be negative at the location where the CpG sites are sparse. **(C)** Top 10 significantly enriched gene ontology (GO) terms of the DMGs for KBD vs. Controls, and OA vs. Controls. The size of dots represents the *q-values* of the GO, and the color of the blocks represents the ratio of genes enriched in the GO terms. PMD, the absolute percent methylation difference.

The third analysis was performed to examine the crucial role of regulation mechanism of methylation regions associated with expressed genes. To do so, we inferred the detected DMRs to the genes covered by the array. With no stringent criterion, the associated DMRs of KBD vs. Controls (including 813 hypermethylated and 1,549 hypomethylated), OA vs. Controls (including 437 hypermethylated and 448 hypomethylated) were 2,362 genes and 885 genes, and 386 genes (KBD vs. OA, including 285 hypermethylated genes and 101 hypomethylated genes), respectively (Supplementary Figure 5B). Compared with OA vs. Control, more CpG sites were differentially methylated with KBD vs. Control. As a consequence, more genes were involved. Our results were largely consistent with previous studies (Duan et al., 2010; Wang et al., 2017). This was because OA was a classic age-related disorder (Anderson and Loeser, 2010; Loeser, 2017), while epigenetic factors largely contributed to the development of KBD (Lü et al., 2011). With the stringent criterion, the associated DMRs of KBD vs. Controls (including 51 hypermethylated and 59 hypomethylated), OA vs. Controls (including 14 hypermethylated and 31 hypomethylated), and KBD vs. OA were 110 genes, 45 genes, and three genes, respectively (Figure 1C). A total of 75 unique DMGs were associated with KBD, while 10 unique DMGs were associated with OA, 35 genes were overlapped between KBD and OA (Jaccard index = 0.291; Fisher's exact test: *p* − *value* < 2.2 × 10^−16^) and one gene overlapping between KBD samples and KBD vs OA samples (Jaccard index = 0.009; Fisher's exact test: *p* − *value* = 2.2 × 10^−2^; Figure 2C). All of the common DMGs shared the same methylation direction in KBD vs. Control and OA vs. Control, implying the similar pathogenic mechanism of KBD and OA. Among those common shared 35 DMGs, Homebox (HOX) family genes play a key role in regulating the chondrogenesis and, therefore, was required for chondrocyte proliferation (Yu et al., 2007); GATA3 was a crucial gene regulating the chondrocyte differentiation contributed to both KBD (Wang et al., 2017) and OA (Goldring, 2012). For KBD-specific DMGs, *HDAC4* is a crucial gene that regulates growth plate chondrocyte differentiation (Chen et al., 2016), while one of the basic pathological feature of KBD is a focal necrosis of chondrocytes in the hypertrophic zone of growth plate cartilage (Ren et al., 2007). Another example is *SMOC2*, which plays an important role in bone mineralization, cell–matrix interactions, collagen binding, and bone remodeling (Brekken and Sage, 2000). The DMRs harbored in *HDAC4* and *SMOC2* are shown in Figures 2A,B. Besides, the KBD-specific DMGs, the OA-specific DMGs, and *SULF1* encodes the enzyme Sulf1, which helps in establishing GAG sulfation in the endoplasmic reticulum and also regulates *Wnt* signaling through desulfation of cell surface HSPGs (Dhoot et al., 2001), while OA is associated with changes in GAG expression levels, sulfation patterns (Sauerland et al., 2003), and *Wnt* signaling pathway (Wang et al., 2019). A previous study also showed that *SULF1* expressed significantly higher in the OA cartilage compared with normal human articular cartilage (Otsuki et al., 2008). All 14 putative marker genes with strong evidence for KBD and OA are shown in Table 2.

**Table 2:** Kashin–Beck disease (KBD) and osteoarthritis (OA) associated differentially methylated genes (DMGs).

	**Gene symbol**	**Function**	**Methylation status**
Common Shared DMGs	SHOX2	Acts upstream the Runx2, the key regulator of chondrogenesis (Cobb et al., 2006)	Hypermethylated
	HOXD3, HOXA13, HOXC9	Critical genes for various cartilage/skeletal physiologic and pathologic process (Quinonez and Innis, 2014; Song et al., 2020)	Hypermethylated Hypomethylated
	GATA3	Crucial gene regulated the chondrocyte differentiation (Singh et al., 2018)	Hypomethylated
	KIF26B	A critical regulator of pathological ossification, with a putative role in heterotopic ossification pathogenesis (Pickering et al., 2018).	Hypermethylated
	CHST11	Encoding protein carbohydrate sulfotransferase 11 on cartilage extracellular matrix biosynthesis (Reynard et al., 2016).	Hypermethylated
KBD-specific DMGs	COL22A1	A Gene that encoding for Collagen XXII which is an extracellular matrix (ECM) protein localized at the articular cartilage-synovial fluid junction (Koch et al., 2004). Besides, COL22A1 is a negative regulator of chondrocyte hypertrophy through interacting with β1−integrin (Zwolanek et al., 2014)	Hypermethylated
	HDAC4	Crucial gene that regulates growth plate chondrocyte differentiation (Chen et al., 2016)	Hypomethylated
	NFIC	Coding protein nuclear factor I C, which affects chondrocyte proliferation (Lee et al., 2020).	Hypermethylated
	SMOC2	SMOC2 negatively affects chondrogenic differentiation of precursor cells and increases IL-1β induced breakdown in adult cartilage (Peeters et al., 2017).	Hypermethylated
	BOC	With a putative role in facilitating articular chondrocyte specificity for conserved signaling pathways and contributes to receptor complex formation (Vanderman et al., 2011).	Hypermethylated
OA-specific DMGs	SULF1	Encodes the enzyme Sulf1 which helps establishing GAG sulfation in the edoplasmatic reticulum, and also regulates Wnt signaling through desulfation of cell surface HSPGs (Otsuki et al., 2008)	Hypermethylated
	EN1	Rs4144782 located in EN1 was significantly associated with increased risk of knee OA (Li et al., 2017)	Hypermethylated

*The putative marker genes of KBD specific and OA specific DMGs were selected using PMD > 20%. The second column is gene symbol, and the third column is the corresponding functions. PMD, the absolute percent methylation difference. All the common DMGs share the same methylation status (hypermethylated gene or hypomethylated gene) for both KBD and OA*.

Finally, we further performed gene set enrichment analysis (GSEA) of DMGs (details in the *Materials and Methods* section). The KBD-associated DMGs were enriched in a total of 51 unique GO terms and one KEGG pathway (Supplementary Table 4), while the OA-associated DMGs were enriched in a total of 126 unique GO terms and one KEGG pathway (Supplementary Table 5). The KBD- and OA-shared DMGs were mainly enriched in GO terms or KEGG pathways that are directly related to the skeletal system and limb-associated pathways among the GO biological processes including embryonic organ development, skeletal system morphogenesis, limb morphogenesis, chondrocyte development, etc. (Supplementary Tables 4, 5, Figure 2C, and Supplementary Figure 6). Besides, we found that KBD-specific DMGs were enriched in wound response and healing pathways, and coagulation associated pathways (Supplementary Table 4). For example, regulation of response to wounding (GO:1903034) is affected by trace elements (Abou Shady et al., 1991; Chen et al., 2017). These results were consistent with the previous differential gene expression analysis finding that several KBD-associated genes were also associated with wound healing and repair (Duan et al., 2010; Wang et al., 2012). However, OA-specific DMGs were enriched in ion-associated GO molecular functions such as gated channel activity and calcium-activated potassium channel activity, for example, potassium channel activity (GO:0005267) and calcium-activated cation channel activity (GO:0005227). Besides, our results showed that DMGs associated with OA were enriched in several ion channel pathways, which was also consistent with the previous studies that ion channels were potential biomarkers for OA (Lewis and Barrett-Jolley, 2015) (Supplementary Table 5).

## Discussion

In this paper, we performed extensive differentially methylated region (DMR) analysis to reveal the pathogenetic similarities and differences between KBD and OA. We presented the novel differentially methylated signals associated with articular cartilage degeneration in KBD and OA in terms of DMRs, DMGs, and functional pathways. Previous studies mainly focused on differentially methylated site analysis, while this work was preliminarily performing DMR analysis.

Age is usually strongly correlated with changes in DNA methylation. We found that 66% significant KBD-associated DMSs were strongly correlated with age and 100% significant OA-associated DMSs were strongly correlated with age. Further, we found that there were 4,325 DMSs for KBD that were correlated with disease status, while only one DMS for OA was correlated with disease status after removing age effects. These results confirmed that the DNA methylation pattern in OA samples was altered by age but not in KBD.

With controlling the age and sex as covariates, we identified 2.25-fold more KBD-associated DMRs and 1.5-fold more KBD-associated DMGs than the OA-associated DMRs and DMGs. Such results confirmed that epigenetic factors may contribute to KBD more than OA, while age contributed to OA more than KBD. Compared with the previous study, which identified 1,212, and 656 DMSs for KBD vs. Controls, and OA vs. Controls, respectively (Wang et al., 2017), we found that only 212 out of 1,212 DMSs (17.5%) were harbored in the DMRs we identified, and the 103 out of 656 DMSs (15.7%) were harbored in the DMRs we identified. This discrepancy was presumably due to the previous study that did not account for the confounding factors, such as age and sex. In addition, 74 out of 110 DMGs (67.2%) annotated for KBD vs. Controls and 31 out of 45 DMGs (68.9%) annotated for OA vs. Controls were also identified in previous study. Overall, more than 30% DMGs we identified were novel DMGs. Further examination of those non-overlapped DMGs would yield additional insights.

In this paper, we found several potential DMGs to be crucial for KBD. For example, the methylation region overlapped with gene *HDAC4* was significantly differentially methylated between KBD and healthy subjects. *HDAC4* encoded HDAC4 protein, which encode histone deacetylase 4 protein. Previous studies have reported that *HDAC4* not only downregulates a series of cartilage-related gene expressions in human chondrocytes such as *Runx2, MMP-1, MMP-3, MMP-13, ADAMTS4*, and *ADAMTS5* but also partially blocks the catabolic events in chondrocytes stimulated by 1L-1β (Cao et al., 2014). 1L-1β induced cartilage degradation by inducing the expression of *MMP-1, MMP-13* (Lee et al., 2012), and aggrecanase-1/-2 (Sylvester et al., 2012) in chondrocytes and synovial fibroblasts. Further, HADC4 is a direct target of microRNA miR-365, which could repress chondrocyte differentiation in growth plate chondrocytes by targeting the 3′UTR of HDAC4 mRNA in osteoblasts (Yang et al., 2016; Xu et al., 2017). DMR analysis and previous studies suggested that the role of *HDAC4* in KBD was crucial as it might serve as a potential drug target for clinical therapy.

Another example is *SMOC2* that encodes SMOC2 protein, which belongs to the SPARC family (selected protein acidic and rich in cysteine/osteonectin/BM-40). SPARC is then matricellular proteins highly expressed in bone matrix that can modulate both matrix metalloprotease (MMP) and growth factor activates (Alford and Hankenson, 2006). Therefore, *SMOC2* plays an important role in bone mineralization, cell–matrix interactions, collagen binding, and bone remodeling (Brekken and Sage, 2000). The SPARC family consists of several proteins, including *SMOCs* (secreted modular calcium-binding proteins), which are extracellular glycoproteins of the SPARC-related modular calcium-binding protein family (Gao et al., 2019). *SMOC2* was observed that it negatively affects chondrogenic differentiation of precursor cells (Peeters et al., 2017). In addition, a positive correlation was observed between *SMOC2* and 1L-1β. However, it may still lack a full understanding of the molecular interactions between *SMOC2* and KBD, as well as how it affected KBD progression. Therefore, further understanding of *SMOC2* is needed to better understand how *SMOC2* affects KBD clinical outcome.

There are also several limitations and potential extensions for this study. First, articular cartilage specimens were collected from five KBD patients, five OA patients, and five healthy subjects for DMR analysis due to the limited number of KBD patients who need surgical treatment. Second, in addition to age and sex, DNA methylation patterns are also influenced by other potential factors such as body mass index (BMI). However, due to the small sample size, we did not consider other confounding factors in our model, which would cause a substantial loss of degree of freedom. Therefore, some other factors would remain probable confounding factors for the DMR analysis. Third, we only performed differentially methylated region analysis for KBD and OA, while integrative analysis of DNA methylation data and gene expression data from the same individuals has become an increasingly common approach in DNA methylation analysis (Hotta et al., 2018). Because the existing gene expression data (Wang et al., 2009; Duan et al., 2010) and DNA methylation data were not matched with the same individuals, we did not attempt to extend our analysis to gene expression data. Nevertheless, measuring the DNA methylation and gene expression data from the same individuals and performing integrating analysis is an important future direction. Finally, our analysis did not account for cellular heterogeneity present in microarray data. Indeed, articular cartilage is a physiologically non-self-renewing avascular tissue, consisting of only chondrocytes (Jiang and Tuan, 2015). Chondrocytes could be further classified to several articular cartilage chondrocyte subtypes (St-Jacques et al., 1999; Saito et al., 2010; Prein et al., 2016). Therefore, the cellular composition of cartilage samples is heterogeneous and varying. Failed accounting for the cellular heterogeneity would affect the accuracy of the methylation–disease association estimation.

In conclusion, we have presented a comprehensive DMR analysis for KBD vs. health controls, OA vs. health controls, and identified a group of DMRs and DMGs for KBD and OA. To the best of our knowledge, this is also the first DMR analysis for KBD and OA, and provides new insights about the similarities and differences between KBD and OA at the methylation level. We hope that our results could provide novel clues for better understanding the pathogenesis and diagnosis for KBD cartilage disease.

## Data Availability Statement

The original contributions presented in the study are included in the article/Supplementary Material, further inquiries can be directed to the corresponding author/s.

## Ethics Statement

This study was approved by the Institutional Review Board (IRB) of Xi'an Jiaotong University. All subjects gave their informed written consent by signing a document that had been carefully reviewed by the IRB of Xi'an Jiaotong University.

## Author Contributions

SS conceived the idea, provided funding support, and designed the experiments. YF performed the real data analyses. FZ and YW provided the real data set. YF, DG, YW, JZ, FZ, LW, and XG revised the manuscript. SS and YF wrote the manuscript. All authors contributed to the article and approved the submitted version.

## Conflict of Interest

The authors declare that the research was conducted in the absence of any commercial or financial relationships that could be construed as a potential conflict of interest.
