# Supplementary material for: Genome-Wide Differentially Methylated Region Analysis to Reveal Epigenetic Differences of Articular Cartilage in Kashin–Beck Disease and Osteoarthritis
Source: Front Cell Dev Biol. 2021 Mar 1;9:636291. doi: 10.3389/fcell.2021.636291 (PMC7957013; doi:10.3389/fcell.2021.636291)
Supplement: Supplementary file 1 [file Table_1.DOCX]

**Supplementary Figure 1**. **The histograms plots to show the distribution of both *β*-value and *M*-value measurements.**(**A**) *β*-value measurement for DNA methylation levels across all analyzed CpG sites. The ***β*-**values show a bimodal distribution between 0 and 1; (**B**) M-value measurement for DNA methylation levels, distributed around -5 to 5. The $M-value$ shows the larger difference than*β*-value, may provide more insight into the methylation level difference between case and control.


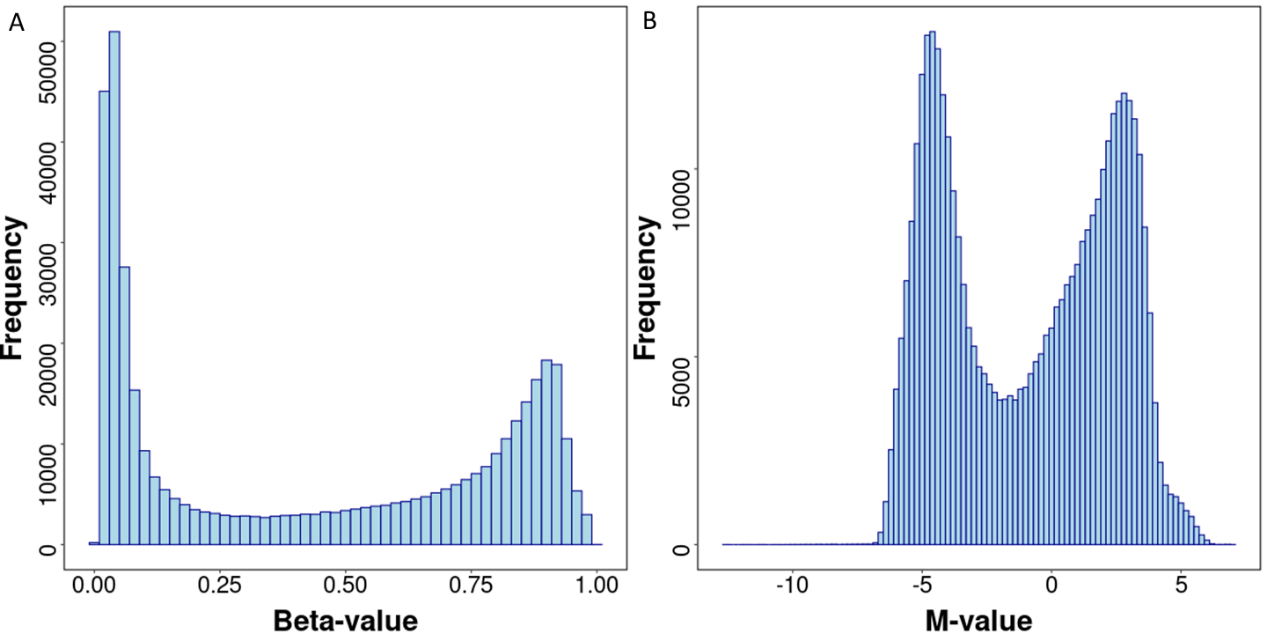


**Supplementary Figure 2**. The scatter plot shows the correlation between the first PC of methylation level of CpG sites which are either significant in (A)KBD samples or in (B)OA samples and the corresponding ages. The correlation between the PC and ages were significant higher in OA samples than in KBD samples.


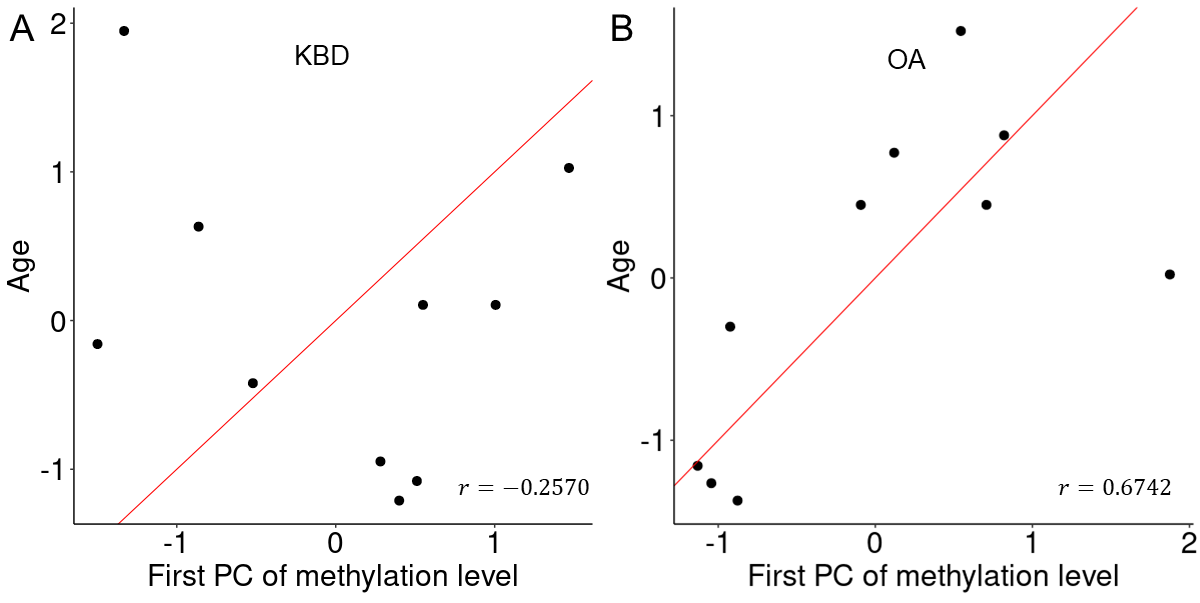


**Supplementary Figure 3**. Top 10 methylation principal components based on M values to examined the batch differences among KBD, OA and Control samples. Heatmap showed that the batch differences among KBD, OA and Control samples were not significant.


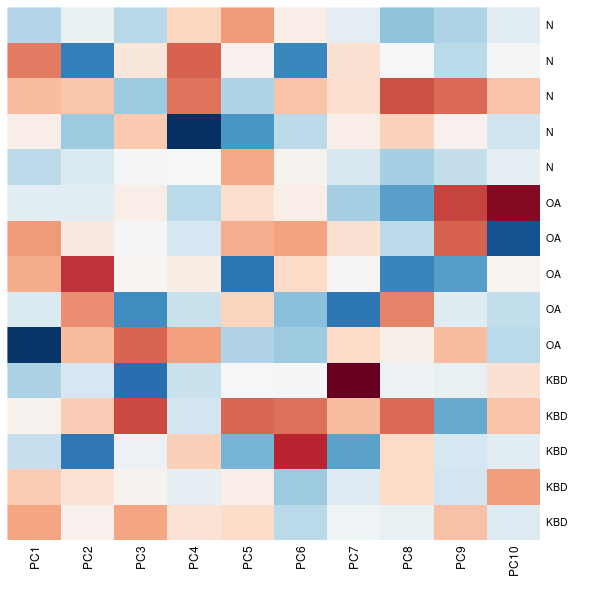


**Supplementary Figure 4**. The bubble plot to show CpG sites harbored in the DMRs of KBD vs. OA. The distribution of CpG sites was similar comparing with KBD vs. Control and OA vs. Control.

**
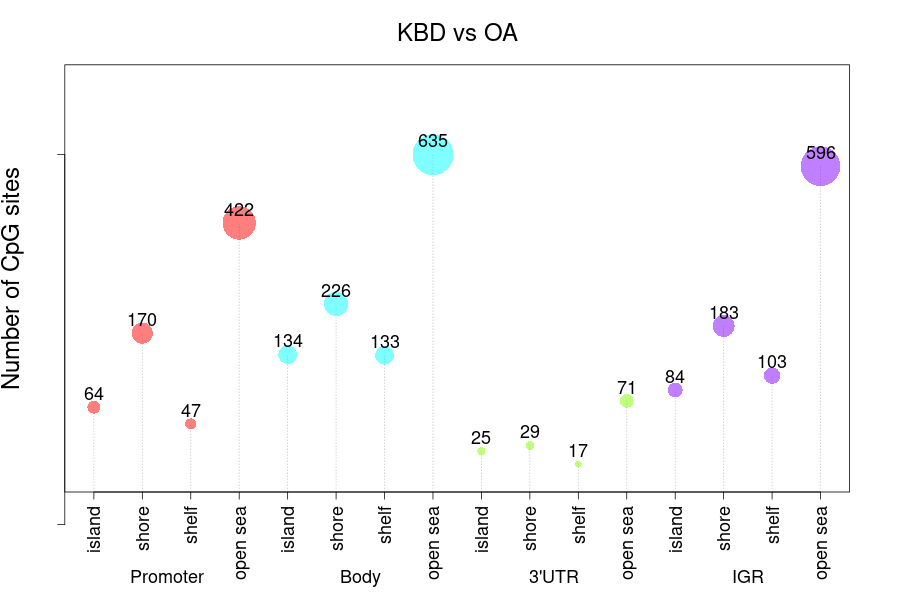
**

**Supplementary Figure 5**. **Venn diagram plots show the overlap of differentially methylated regions and differentially methylated genes between KBD and OA**. (**A**) The overlapping of DMRs between KBD vs. Control and OA vs. Control. We defined the regions are overlapped if the width of the overlapped part exceeds 50% comparing with the narrower region. (**B**) the overlapping of its corresponding DMR associated DMGs between KBD vs. Controls and OA vs. Controls.


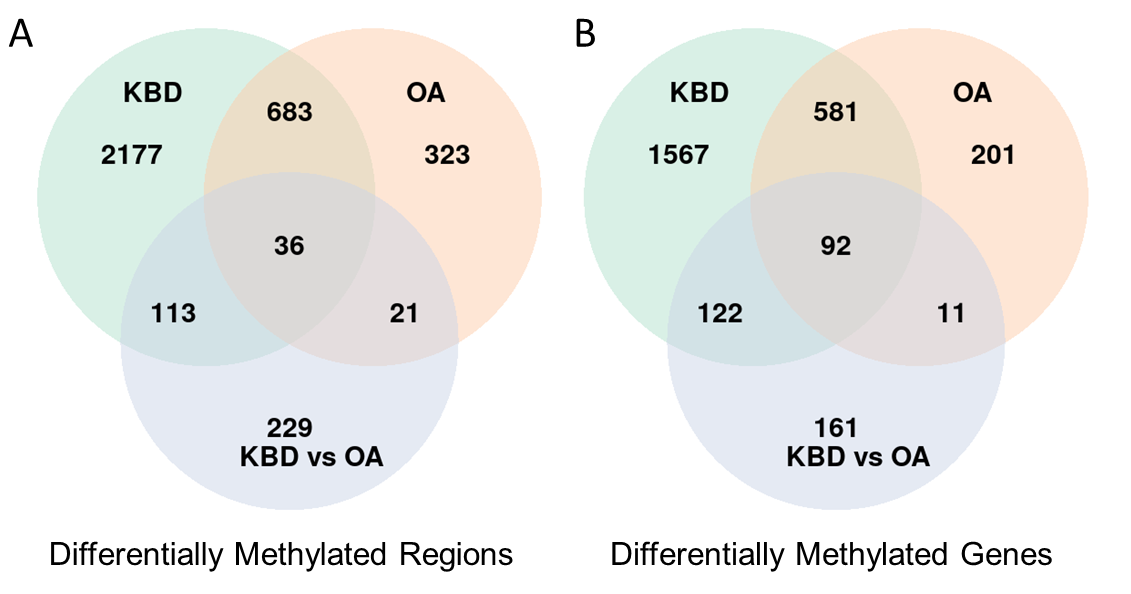


**Supplementary Figure 6.**  Both KBD and OA associated DMGs are enriched in Glycosaminoglycan biosynthesis – chondroitin sulfate / dermatan sulfate pathway. Chondroitin sulfate and dermatan sulfate are sulfated linear polysaccharides, which are synthesized as sidechains of proteoglycans (PGs) and are found in the extracellular matrix (ECM) or bound to cell surfaces


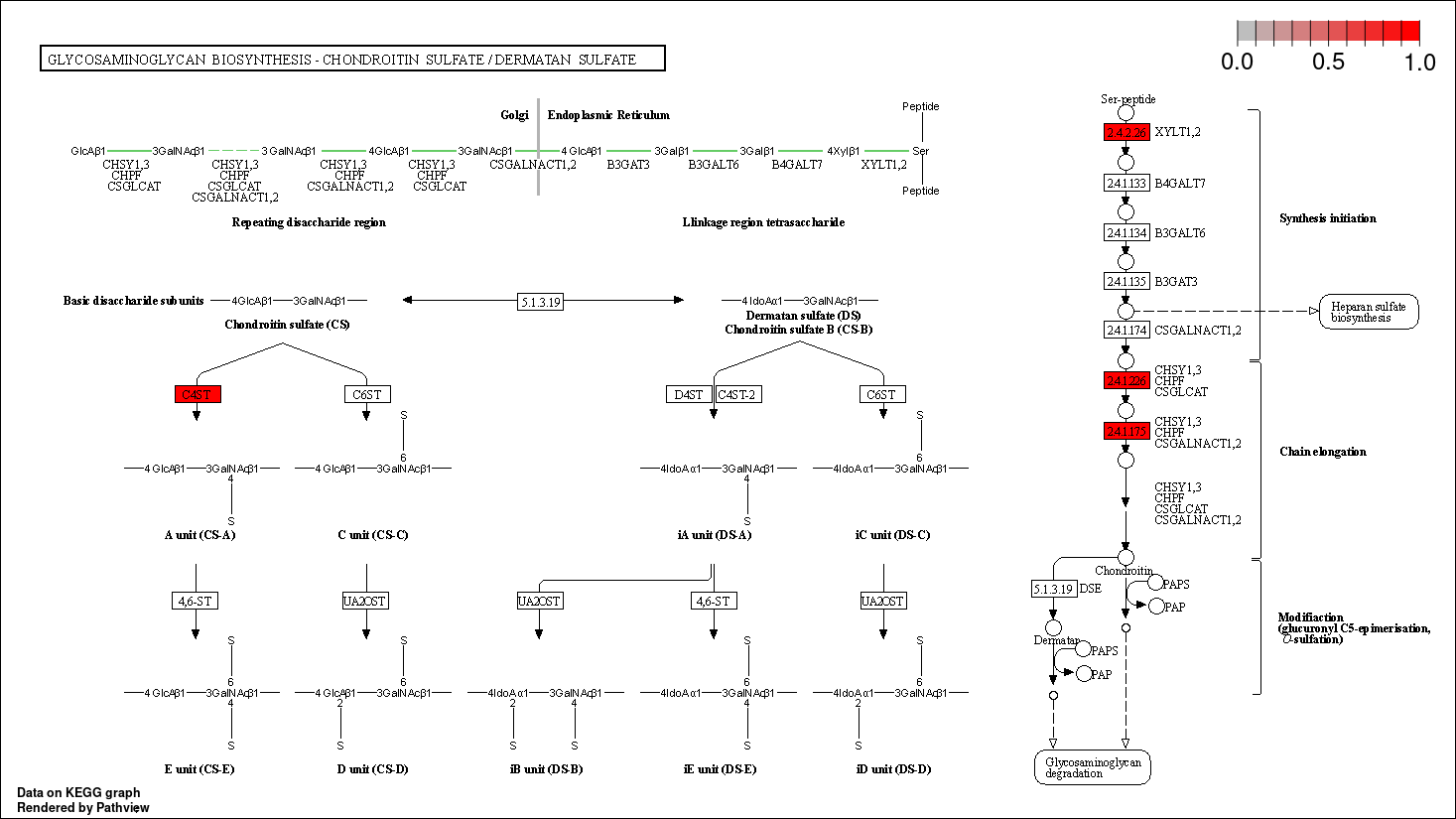


**Supplementary Table 1**. **Differentially methylated DMRs with large PMD (i.e >20%) between KBD and normal patients**. The PMD is defined as the difference of the average percent methylation level between regions. Data are sorted in rows based on the adjust *p-value*.

| Chromosome | Start | End | Width | *p-value* | Annotated gene | PMD | #CpG sites |
| --- | --- | --- | --- | --- | --- | --- | --- |
| chr2 | 177018871 | 177024333 | 5463 | *7.80E-42* | HOXD3 | 0.28579 | 19 |
| chr11 | 15959441 | 15960097 | 657 | *1.97E-25* | SOX6 | 0.374391 | 7 |
| chr2 | 66663710 | 66668496 | 4787 | *5.73E-24* | MEIS1;MEIS1-AS2 | 0.203732 | 20 |
| chr17 | 73055309 | 73056670 | 1362 | *1.19E-19* | KCTD2 | 0.313449 | 6 |
| chr7 | 27240679 | 27244170 | 3492 | *5.60E-18* | NA;HOTTIP | 0.461492 | 9 |
| chr3 | 147105149 | 147107240 | 2092 | *8.25E-18* | ZIC4-AS1;ZIC4 | 0.204167 | 9 |
| chr12 | 1609238 | 1610224 | 987 | *4.76E-17* | ERC1 | 0.215464 | 9 |
| chr10 | 118350351 | 118350866 | 516 | *3.52E-12* | PNLIPRP1 | 0.215135 | 4 |
| chr3 | 112932491 | 112936686 | 4196 | *7.41E-12* | BOC | 0.304198 | 4 |
| chr20 | 39125270 | 39128282 | 3013 | *9.57E-12* | MAFB | 0.384504 | 3 |
| chr3 | 15310537 | 15311753 | 1217 | *1.20E-11* | SH3BP5 | 0.396155 | 4 |
| chr4 | 54424280 | 54425578 | 1299 | *1.69E-11* | LNX1 | 0.203475 | 7 |
| chr9 | 969311 | 969634 | 324 | *3.51E-11* | DMRT1 | 0.389479 | 3 |
| chr7 | 2060034 | 2060325 | 292 | *4.38E-11* | MAD1L1 | 0.273794 | 4 |
| chr1 | 230249482 | 230250840 | 1359 | *4.44E-11* | GALNT2 | 0.205477 | 4 |
| chr3 | 157813165 | 157813831 | 667 | *8.13E-11* | SHOX2 | 0.386254 | 6 |
| chr5 | 1015796 | 1017252 | 1457 | *1.49E-10* | NKD2 | 0.322204 | 5 |
| chr5 | 3605621 | 3607215 | 1595 | *1.61E-10* |  | 0.26229 | 10 |
| chr7 | 101499531 | 101500786 | 1256 | *2.96E-10* |  | 0.305488 | 4 |
| chr12 | 114873892 | 114878254 | 4363 | *3.47E-10* | TBX5 | 0.396839 | 9 |
| chr7 | 26896698 | 26898198 | 1501 | *5.06E-10* | SKAP2 | 0.217858 | 7 |
| chr3 | 178984490 | 178985005 | 516 | *1.90E-09* | KCNMB3 | 0.278176 | 4 |
| chr2 | 68592254 | 68593221 | 968 | *2.55E-09* | PLEK;AC015969.3 | 0.203338 | 4 |
| chr17 | 57918146 | 57919166 | 1021 | *3.38E-09* | VMP1;MIR21 | 0.305322 | 5 |
| chr5 | 2251596 | 2254523 | 2928 | *4.18E-09* | Y_RNA | 0.245235 | 5 |
| chr16 | 17254067 | 17255542 | 1476 | *5.06E-09* | XYLT1 | 0.204511 | 4 |
| chr10 | 1980154 | 1982954 | 2801 | *5.67E-09* | LINC00700 | 0.301287 | 3 |
| chr17 | 77037658 | 77038222 | 565 | *5.67E-09* | C1QTNF1 | 0.317763 | 3 |
| chr8 | 18540962 | 18542110 | 1149 | *5.95E-09* | PSD3 | 0.209386 | 5 |
| chr14 | 54814464 | 54817453 | 2990 | *9.82E-09* | CDKN3 | 0.202257 | 4 |
| chr3 | 124103076 | 124104180 | 1105 | *1.55E-08* | KALRN | 0.206967 | 4 |
| chr7 | 101595647 | 101596888 | 1242 | *1.91E-08* |  | 0.225492 | 5 |
| chr10 | 3535060 | 3538567 | 3508 | *2.28E-08* | RP11-482E14.1 | 0.223564 | 6 |
| chr20 | 57470829 | 57472498 | 1670 | *2.39E-08* | GNAS;HNRNPUL2-BSCL2 | 0.222053 | 5 |
| chr1 | 115238350 | 115238667 | 318 | *5.92E-08* | AMPD1 | 0.216712 | 3 |
| chr19 | 11289204 | 11289522 | 319 | *5.92E-08* | KANK2 | 0.223807 | 5 |
| chr5 | 2866697 | 2866933 | 237 | *6.19E-08* | C5orf38 | 0.211676 | 4 |
| chr8 | 124214470 | 124215858 | 1389 | *8.09E-08* | FAM83A-AS1;FAM83A | 0.246912 | 4 |
| chr2 | 11679622 | 11680278 | 657 | *9.85E-08* | GREB1 | 0.200901 | 6 |
| chr12 | 111402559 | 111405485 | 2927 | *2.05E-07* | LINC01405 | 0.219043 | 3 |
| chr5 | 14405149 | 14407215 | 2067 | *2.16E-07* | TRIO | 0.231324 | 8 |
| chr12 | 115104190 | 115104896 | 707 | *3.33E-07* | TBX3 | 0.222335 | 6 |
| chr2 | 21228737 | 21229806 | 1070 | *3.86E-07* | APOB | 0.230904 | 3 |
| chr6 | 11779803 | 11780018 | 216 | *6.61E-07* | ADTRP | 0.221289 | 3 |
| chr2 | 45233383 | 45233780 | 398 | *6.95E-07* | SIX2 | 0.431307 | 4 |
| chr11 | 16631704 | 16632249 | 546 | *7.89E-07* | RN7SL188P | 0.359767 | 4 |
| chr15 | 101728127 | 101730009 | 1883 | *1.00E-06* | CHSY1 | 0.23378 | 6 |
| chr10 | 79110149 | 79111517 | 1369 | *1.06E-06* | KCNMA1 | 0.214643 | 3 |
| chr6 | 151345785 | 151346892 | 1108 | *1.09E-06* | MTHFD1L | 0.27564 | 3 |
| chr7 | 1433819 | 1436446 | 2628 | *1.11E-06* | MICALL2 | 0.271372 | 3 |
| chr14 | 105436489 | 105437625 | 1137 | *1.13E-06* | AHNAK2 | 0.207835 | 3 |
| chr19 | 38886512 | 38886970 | 459 | *1.69E-06* | SPRED3 | 0.227611 | 4 |
| chr1 | 158979553 | 158980099 | 547 | *2.17E-06* | IFI16 | 0.326353 | 3 |
| chr10 | 106088444 | 106089261 | 818 | *2.17E-06* | ITPRIP | 0.20102 | 5 |
| chr8 | 435411 | 438247 | 2837 | *2.78E-06* | TDRP | 0.217107 | 3 |
| chr10 | 11317304 | 11318356 | 1053 | *5.04E-06* | CELF2 | 0.237686 | 3 |
| chr11 | 628515 | 628838 | 324 | *5.99E-06* | NA | 0.270078 | 3 |
| chr10 | 99797114 | 99799712 | 2599 | *6.77E-06* | CRTAC1 | 0.318232 | 3 |
| chr5 | 4114890 | 4117865 | 2976 | *7.19E-06* | NA | 0.214944 | 5 |
| chr15 | 88575812 | 88576804 | 993 | *1.05E-05* | NTRK3 | 0.332098 | 3 |
| chr3 | 169381547 | 169385780 | 4234 | *1.07E-05* | NA | 0.207987 | 9 |
| chr2 | 240230103 | 240231375 | 1273 | *1.27E-05* | HDAC4 | 0.295776 | 4 |
| chr4 | 187563508 | 187564475 | 968 | *1.29E-05* | FAT1 | 0.254482 | 3 |
| chr15 | 37180621 | 37180944 | 324 | *1.32E-05* | MEIS2 | 0.238344 | 3 |
| chr2 | 235862496 | 235865687 | 3192 | *1.45E-05* | SH3BP4 | 0.241383 | 3 |
| chr16 | 51168403 | 51168635 | 233 | *1.78E-05* | SALL1 | 0.350758 | 3 |
| chr12 | 25150500 | 25150733 | 234 | *1.88E-05* | C12orf77 | 0.327141 | 3 |
| chr11 | 314936 | 315444 | 509 | *2.53E-05* | IFITM1 | 0.202534 | 3 |
| chr11 | 33744309 | 33745081 | 773 | *2.97E-05* | CD59 | 0.236669 | 4 |
| chr10 | 134376860 | 134377965 | 1106 | *4.00E-05* | INPP5A | 0.285185 | 3 |
| chr5 | 134526011 | 134526430 | 420 | *4.08E-05* | C5orf66 | 0.377578 | 3 |
| chr7 | 23386882 | 23388345 | 1464 | *4.20E-05* | IGF2BP3 | 0.301876 | 4 |
| chr5 | 3592382 | 3592607 | 226 | *5.13E-05* |  | 0.217898 | 3 |
| chr1 | 245411100 | 245412195 | 1096 | *5.16E-05* | KIF26B | 0.342849 | 3 |
| chr7 | 944607 | 944898 | 292 | *8.33E-05* | COX19;ADAP1 | 0.262472 | 3 |
| chr20 | 62318348 | 62318560 | 213 | *0.000113* | RTEL1-TNFRSF6B | 0.210613 | 3 |
| chr6 | 169688492 | 169691221 | 2730 | *0.000148* | XXyac-YX65C7_A.3 | 0.269223 | 3 |
| chr16 | 48180293 | 48180921 | 629 | *0.000164* | ABCC12 | 0.320857 | 3 |
| chr10 | 130544927 | 130547580 | 2654 | *0.000164* |  | 0.216442 | 3 |
| chr19 | 3464859 | 3465233 | 375 | *0.000209* | NFIC | 0.208346 | 4 |
| chr19 | 7580154 | 7580279 | 126 | *0.000243* | ZNF358 | 0.25057 | 3 |
| chr15 | 70765902 | 70768930 | 3029 | *0.000249* | UACA | 0.272505 | 4 |
| chr2 | 238646458 | 238648738 | 2281 | *0.000262* | LRRFIP1 | 0.215938 | 4 |
| chr10 | 28525029 | 28526238 | 1210 | *0.000290* | MPP7 | 0.2069 | 3 |
| chr19 | 31640668 | 31640735 | 68 | *0.000308* | TSHZ3 | 0.284739 | 3 |
| chr10 | 70847242 | 70847538 | 297 | *0.000329* | SRGN | 0.218912 | 3 |
| chr8 | 139783552 | 139784933 | 1382 | *0.000332* | COL22A1 | 0.253725 | 3 |
| chr6 | 41438538 | 41438861 | 324 | *0.000440* | FOXP4-AS1 | 0.237846 | 3 |
| chr10 | 77794107 | 77795180 | 1074 | *0.000449* | C10orf11 | 0.210044 | 3 |
| chr2 | 232219267 | 232222958 | 3692 | *0.000556* | ARMC9 | 0.205349 | 3 |
| chr14 | 103294105 | 103294997 | 893 | *0.000728* | TRAF3 | 0.230156 | 5 |
| chr8 | 216578 | 216741 | 164 | *0.000771* | ZNF596 | 0.210112 | 3 |
| chr16 | 54610283 | 54613004 | 2722 | *0.000815* |  | 0.221302 | 3 |
| chr12 | 105113679 | 105114882 | 1204 | *0.000952* | CHST11 | 0.228801 | 3 |
| chr12 | 54389730 | 54390144 | 415 | *0.001106* | HOXC9 | 0.383011 | 3 |
| chr12 | 111664099 | 111665268 | 1170 | *0.001296* | CUX2 | 0.211426 | 6 |
| chr2 | 10301907 | 10302946 | 1040 | *0.001382* | C2orf48 | 0.230502 | 3 |
| chr19 | 49935815 | 49935972 | 158 | *0.001439* | SLC17A7 | 0.229978 | 3 |
| chr1 | 9400435 | 9401033 | 599 | *0.001590* | SPSB1 | 0.24442 | 3 |
| chr7 | 27235923 | 27237424 | 1502 | *0.001673* | NA;HOXA13 | 0.314455 | 3 |
| chr8 | 37308529 | 37311127 | 2599 | *0.001765* | RNU6-607P | 0.226164 | 3 |
| chr7 | 27233249 | 27233641 | 393 | *0.002251* |  | 0.537925 | 3 |
| chr17 | 17714275 | 17714418 | 144 | *0.002303* | RAI1 | 0.206618 | 3 |
| chr16 | 73090607 | 73090838 | 232 | *0.00247* | ZFHX3 | 0.222035 | 3 |
| chr6 | 169001470 | 169002604 | 1135 | *0.002534* | SMOC2 | 0.282588 | 3 |
| chr10 | 8124317 | 8127052 | 2736 | *0.002759* | GATA3 | 0.279174 | 3 |
| chr15 | 39874829 | 39877073 | 2245 | *0.002782* | THBS1 | 0.233283 | 3 |
| chr7 | 27228900 | 27229324 | 425 | *0.003397* |  | 0.357209 | 3 |
| chr1 | 234851515 | 234854173 | 2659 | *0.003676* |  | 0.271755 | 3 |
| chr2 | 46592740 | 46593755 | 1016 | *0.004158* | EPAS1 | 0.266788 | 3 |
| chr7 | 630327 | 630726 | 400 | *0.004707* | PRKAR1B | 0.20478 | 3 |
| chr6 | 130181570 | 130182727 | 1158 | *0.004985* | TMEM244 | 0.222121 | 7 |
| chr6 | 168925150 | 168926181 | 1032 | *0.005928* | SMOC2 | 0.221524 | 3 |
| chr12 | 114887297 | 114890788 | 3492 | *0.006060* | TBX5 | 0.208568 | 3 |
| chr16 | 86598630 | 86599049 | 420 | *0.006160* | FOXC2 | 0.22505 | 3 |
| chr10 | 119310127 | 119310565 | 439 | *0.007389* | EMX2 | 0.239942 | 3 |
| chr16 | 70759430 | 70760625 | 1196 | *0.007532* | VAC14 | 0.201966 | 3 |
| chr7 | 28837602 | 28838717 | 1116 | *0.007648* | CREB5 | 0.216659 | 3 |
| chr21 | 43619451 | 43619666 | 216 | *0.008095* | ABCG1 | 0.214609 | 3 |
| chr2 | 177030035 | 177030344 | 310 | *0.008359* | HOXD3 | 0.200002 | 3 |
| chr10 | 79421022 | 79423673 | 2652 | *0.012267* | KCNMA1 | 0.447126 | 3 |
| chr10 | 6213532 | 6214562 | 1031 | *0.014692* | PFKFB3 | 0.248854 | 3 |
| chr10 | 118568197 | 118570890 | 2694 | *0.014735* | RP11-539I5.1 | 0.209168 | 3 |
| chr13 | 27294647 | 27297292 | 2646 | *0.016267* | WASF3 | 0.261162 | 3 |
| chr6 | 136914604 | 136916040 | 1437 | *0.017139* | MAP3K5 | 0.205727 | 3 |
| chr2 | 209029583 | 209029798 | 216 | *0.018412* | C2orf80 | 0.200708 | 3 |
| chr17 | 38716319 | 38717758 | 1440 | *0.018457* | CCR7 | 0.223963 | 4 |
| chr2 | 176936296 | 176936518 | 223 | *0.019204* | EVX2 | 0.265674 | 3 |
| chr16 | 87172922 | 87175905 | 2984 | *0.026392* | RP11-178L8.1 | 0.211109 | 3 |
| chr7 | 1135877 | 1136168 | 292 | *0.040238* | C7orf50 | 0.205713 | 3 |
| chr2 | 239169054 | 239170179 | 1126 | *0.046577* | PER2 | 0.219583 | 3 |

**Supplementary Table 2. Differentially methylated DMRs with large PMD (i.e >20%) between OA and normal patients**. The PMD is defined as the difference of the average percent methylation level between regions. Data are sorted in rows based on the adjust *p-value*

| Chromosome | Start | End | Width | *p-value* | Annotated gene | PMD | #CpG sites |
| --- | --- | --- | --- | --- | --- | --- | --- |
| chr2 | 177018871 | 177024333 | 5463 | *1.34E-36* | HOXD3 | 0.267727 | 19 |
| chr11 | 15959441 | 15960097 | 657 | *5.71E-19* | SOX6 | 0.412999 | 7 |
| chr7 | 27240679 | 27244170 | 3492 | *2.71E-16* | HOTTIP | 0.476266 | 9 |
| chr4 | 154125237 | 154126204 | 968 | *2.48E-13* | RP11-335O4.3;TRIM2 | 0.219243 | 7 |
| chr5 | 3605621 | 3607215 | 1595 | *2.04E-10* |  | 0.229615 | 10 |
| chr2 | 119602401 | 119602980 | 580 | *5.60E-10* | EN1 | 0.206544 | 6 |
| chr15 | 101728127 | 101730009 | 1883 | *1.01E-09* | CHSY1 | 0.287446 | 6 |
| chr5 | 1015796 | 1017252 | 1457 | *3.93E-09* | NKD2 | 0.31214 | 5 |
| chr5 | 2866697 | 2866933 | 237 | *1.02E-08* | C5orf38 | 0.300366 | 4 |
| chr3 | 157813165 | 157813831 | 667 | *3.04E-08* | SHOX2 | 0.402417 | 6 |
| chr2 | 119599662 | 119600665 | 1004 | *6.11E-08* | EN1 | 0.24759 | 9 |
| chr9 | 969311 | 969634 | 324 | *1.20E-07* | DMRT1 | 0.426111 | 3 |
| chr1 | 230249482 | 230250840 | 1359 | *1.79E-07* | GALNT2 | 0.217529 | 4 |
| chr12 | 114873892 | 114878254 | 4363 | *2.18E-07* | TBX5 | 0.388374 | 9 |
| chr17 | 73055309 | 73056670 | 1362 | *6.35E-07* | KCTD2 | 0.236349 | 6 |
| chr10 | 135343048 | 135343425 | 378 | *7.65E-07* | CYP2E1;SPRN | 0.222292 | 3 |
| chr3 | 178984490 | 178985005 | 516 | *1.77E-06* | KCNMB3 | 0.314017 | 4 |
| chr7 | 1433819 | 1436446 | 2628 | *2.92E-06* | MICALL2 | 0.294444 | 3 |
| chr12 | 111402559 | 111405485 | 2927 | *3.96E-06* | LINC01405 | 0.208717 | 3 |
| chr12 | 115104190 | 115104896 | 707 | *4.49E-06* | TBX3 | 0.23519 | 6 |
| chr7 | 101499531 | 101500786 | 1256 | *8.86E-06* |  | 0.294538 | 4 |
| chr5 | 2251596 | 2254523 | 2928 | *1.07E-05* | Y_RNA | 0.217093 | 5 |
| chr5 | 134526011 | 134526430 | 420 | *1.70E-05* | C5orf66 | 0.385354 | 3 |
| chr2 | 119588099 | 119592269 | 4171 | *2.09E-05* | EN1 | 0.237842 | 6 |
| chr20 | 39125270 | 39128282 | 3013 | *2.13E-05* | MAFB | 0.346546 | 3 |
| chr16 | 72458313 | 72461396 | 3084 | *2.55E-05* |  | 0.203084 | 6 |
| chr1 | 245411100 | 245412195 | 1096 | *2.80E-05* | KIF26B | 0.372574 | 3 |
| chr6 | 151345785 | 151346892 | 1108 | *3.68E-05* | MTHFD1L | 0.262804 | 3 |
| chr7 | 2060034 | 2060325 | 292 | *6.25E-05* | MAD1L1 | 0.200323 | 4 |
| chr10 | 1980154 | 1982954 | 2801 | *8.75E-05* | LINC00700 | 0.345001 | 3 |
| chr2 | 191764 | 195771 | 4008 | *8.75E-05* | AC079779.7 | 0.216101 | 4 |
| chr2 | 177039265 | 177039588 | 324 | *0.000105* | HAGLR | 0.210253 | 3 |
| chr15 | 37180621 | 37180944 | 324 | *0.000175* | MEIS2 | 0.223697 | 3 |
| chr6 | 101846790 | 101847801 | 1012 | *0.000181* | GRIK2 | 0.205715 | 14 |
| chr7 | 26896698 | 26898198 | 1501 | *0.000229* | SKAP2 | 0.22022 | 7 |
| chr2 | 45233383 | 45233780 | 398 | *0.00035* | SIX2 | 0.412835 | 4 |
| chr16 | 51168403 | 51168635 | 233 | *0.00036* | SALL1 | 0.349081 | 3 |
| chr12 | 111664099 | 111665268 | 1170 | *0.000813* | CUX2 | 0.241889 | 6 |
| chr10 | 134376860 | 134377965 | 1106 | *0.00103* | INPP5A | 0.267032 | 3 |
| chr13 | 114837551 | 114837842 | 292 | *0.001052* | RASA3 | 0.206822 | 3 |
| chr7 | 27233249 | 27233641 | 393 | *0.00218* |  | 0.585481 | 3 |
| chr10 | 8124317 | 8127052 | 2736 | *0.00237* | GATA3 | 0.254036 | 3 |
| chr7 | 630327 | 630726 | 400 | *0.00237* | PRKAR1B | 0.232742 | 3 |
| chr3 | 15310537 | 15311753 | 1217 | *0.002698* | SH3BP5 | 0.283925 | 4 |
| chr7 | 27228900 | 27229324 | 425 | *0.002754* |  | 0.377364 | 3 |
| chr12 | 54389730 | 54390144 | 415 | *0.003375* | HOXC9 | 0.360542 | 3 |
| chr11 | 16631704 | 16632249 | 546 | *0.004737* | RN7SL188P | 0.254668 | 4 |
| chr8 | 18540962 | 18542110 | 1149 | *0.0082* | PSD3 | 0.200513 | 5 |
| chr10 | 79421022 | 79423673 | 2652 | *0.009299* | KCNMA1 | 0.352963 | 3 |
| chr12 | 105113679 | 105114882 | 1204 | *0.009588* | CHST11 | 0.276925 | 3 |
| chr8 | 70378537 | 70378752 | 216 | *0.009818* | SULF1 | 0.228651 | 3 |
| chr12 | 114887297 | 114890788 | 3492 | *0.010666* | TBX5 | 0.272873 | 3 |
| chr2 | 236281804 | 236284555 | 2752 | *0.010972* |  | 0.20565 | 3 |
| chr7 | 27235923 | 27237424 | 1502 | *0.011039* | HOXA13 | 0.319818 | 3 |
| chr19 | 31640668 | 31640735 | 68 | *0.016168* | TSHZ3 | 0.269263 | 3 |
| chr15 | 32963671 | 32965242 | 1572 | *0.024161* | RP11-1000B6.2;SCG5 | 0.231973 | 6 |
| chr16 | 87098675 | 87103972 | 5298 | *0.033769* | RP11-178L8.1 | 0.229912 | 16 |
| chr10 | 99797114 | 99799712 | 2599 | *0.043157* | CRTAC1 | 0.25023 | 3 |

**Supplementary Table 3. Differentially methylated DMRs with large PMD (i.e >20%) between KBD and OA patients**. The PMD is defined as the difference of the average percent methylation level between regions. Data are sorted in rows based on the adjust *p-value*

| Chromosome | Start | End | Width | *p-value* | Annotated gene | PMD | #CpG sites |
| --- | --- | --- | --- | --- | --- | --- | --- |
| chr5 | 135415109 | 135416412 | 1304 | *5.08E-09* |  | 0.237653 | 15 |
| chr11 | 70563761 | 70563918 | 158 | *0.000298* | SHANK2 | 0.204388 | 3 |
| chr15 | 88575812 | 88576804 | 993 | *0.010792* | NTRK3 | 0.316501 | 3 |
| chr10 | 72453492 | 72454510 | 1019 | *0.032123* | ADAMTS14 | 0.226201 | 3 |

**Supplementary Table 4.** **Significantly enriched GO terms and KEGG pathway from the DMG of KBD vs Control.** Data are sorted in rows based on the *q-value*. The KBD-associated DMGs were significantly enriched in skeletal system and limb associated pathways. We defined the fold change as the average PMD of DMGs involved in the pathway.

| GO Names | Ontology | Description | *p-value* | *q value* | #Genes | Shared | Fold change |
| --- | --- | --- | --- | --- | --- | --- | --- |
| BP | GO:0001501 | skeletal system development | 4.41E-08 | 9.25E-05 | 16 | Shared | 2.40 |
| BP | GO:0048706 | embryonic skeletal system development | 7.34E-08 | 9.25E-05 | 9 | Shared | 1.65 |
| BP | GO:0048704 | embryonic skeletal system morphogenesis | 1.05E-07 | 9.25E-05 | 8 | Shared | 1.44 |
| BP | GO:0048562 | embryonic organ morphogenesis | 1.54E-07 | 0.000101 | 12 | Shared | 0.65 |
| BP | GO:0048568 | embryonic organ development | 1.47E-06 | 0.000772 | 13 | Shared | 0.92 |
| BP | GO:0035107 | appendage morphogenesis | 2.65E-06 | 0.000995 | 8 | Shared | 0.87 |
| BP | GO:0035108 | limb morphogenesis | 2.65E-06 | 0.000995 | 8 | Shared | 0.87 |
| BP | GO:0048701 | embryonic cranial skeleton morphogenesis | 7.70E-06 | 0.002158 | 5 | Shared | 1.16 |
| BP | GO:0030326 | embryonic limb morphogenesis | 9.50E-06 | 0.002158 | 7 | Shared | 1.13 |
| BP | GO:0035113 | embryonic appendage morphogenesis | 9.50E-06 | 0.002158 | 7 | Shared | 1.13 |
| MF | GO:0001228 | DNA-binding transcription activator activity, RNA polymerase II-specific | 1.05E-05 | 0.002158 | 12 | Shared | 1.22 |
| BP | GO:0048736 | appendage development | 1.07E-05 | 0.002158 | 8 | Shared | 0.87 |
| BP | GO:0060173 | limb development | 1.07E-05 | 0.002158 | 8 | Shared | 0.87 |
| MF | GO:0001227 | DNA-binding transcription repressor activity, RNA polymerase II-specific | 1.27E-05 | 0.002394 | 9 | Shared | -0.34 |
| BP | GO:0048705 | skeletal system morphogenesis | 1.36E-05 | 0.002395 | 9 | Shared | 1.68 |
| BP | GO:0048703 | embryonic viscerocranium morphogenesis | 2.75E-05 | 0.004523 | 3 | Shared | 0.73 |
| BP | GO:0035115 | embryonic forelimb morphogenesis | 4.51E-05 | 0.006978 | 4 | Shared | 1.26 |
| BP | GO:1904888 | cranial skeletal system development | 4.90E-05 | 0.007161 | 5 | Shared | 1.16 |
| BP | GO:1903036 | positive regulation of response to wounding | 5.68E-05 | 0.007746 | 5 | KBD-specific | -0.10 |
| BP | GO:0030099 | myeloid cell differentiation | 5.88E-05 | 0.007746 | 10 | KBD-specific | -0.10 |
| BP | GO:0072283 | metanephric renal vesicle morphogenesis | 8.19E-05 | 0.010266 | 3 | Shared | 0.42 |
| BP | GO:0035136 | forelimb morphogenesis | 9.97E-05 | 0.011927 | 4 | Shared | 1.26 |
| BP | GO:0035850 | epithelial cell differentiation involved in kidney development | 0.000122 | 0.013912 | 4 | Shared | 0.03 |
| BP | GO:0060986 | endocrine hormone secretion | 0.000134 | 0.014666 | 4 | KBD-specific | -0.37 |
| BP | GO:0072077 | renal vesicle morphogenesis | 0.000151 | 0.015891 | 3 | Shared | 0.42 |
| BP | GO:0009791 | post-embryonic development | 0.00016 | 0.016235 | 5 | Shared | 0.09 |
| BP | GO:0060231 | mesenchymal to epithelial transition | 0.00018 | 0.016815 | 3 | Shared | -0.20 |
| BP | GO:0072087 | renal vesicle development | 0.00018 | 0.016815 | 3 | Shared | 0.42 |
| BP | GO:0061448 | connective tissue development | 0.000185 | 0.016815 | 8 | Shared | 2.17 |
| BP | GO:0072073 | kidney epithelium development | 0.0002 | 0.017532 | 6 | Shared | 0.15 |
| BP | GO:0001656 | metanephros development | 0.000212 | 0.017532 | 5 | Shared | 0.37 |
| BP | GO:0051216 | cartilage development | 0.000213 | 0.017532 | 7 | Shared | 1.94 |
| BP | GO:0045165 | cell fate commitment | 0.000223 | 0.017792 | 8 | Shared | 1.31 |
| BP | GO:0061005 | cell differentiation involved in kidney development | 0.000265 | 0.020501 | 4 | Shared | 0.03 |
| BP | GO:0001657 | ureteric bud development | 0.000289 | 0.021635 | 5 | Shared | 0.37 |
| BP | GO:0072163 | mesonephric epithelium development | 0.000304 | 0.021635 | 5 | Shared | 0.37 |
| BP | GO:0072164 | mesonephric tubule development | 0.000304 | 0.021635 | 5 | Shared | 0.37 |
| BP | GO:0001708 | cell fate specification | 0.000319 | 0.022127 | 5 | Shared | 0.37 |
| BP | GO:0001823 | mesonephros development | 0.000369 | 0.0249 | 5 | Shared | 1.10 |
| BP | GO:0090303 | positive regulation of wound healing | 0.000383 | 0.025198 | 4 | KBD-specific | 0.3 |
| BP | GO:0030206 | chondroitin sulfate biosynthetic process | 0.000435 | 0.026681 | 3 | Shared | 0.24 |
| BP | GO:0072273 | metanephric nephron morphogenesis | 0.000435 | 0.026681 | 3 | Shared | 0.67 |
| BP | GO:0001503 | ossification | 0.000436 | 0.026681 | 9 | KBD-specific | 0.42 |
| BP | GO:0072009 | nephron epithelium development | 0.000485 | 0.029019 | 5 | Shared | 1.20 |
| BP | GO:0003205 | cardiac chamber development | 0.00055 | 0.031888 | 6 | Shared | 0.37 |
| BP | GO:1903034 | regulation of response to wounding | 0.000568 | 0.031888 | 6 | KBD-specific | 0.60 |
| BP | GO:0060675 | ureteric bud morphogenesis | 0.000569 | 0.031888 | 4 | Shared | -0.41 |
| BP | GO:0072171 | mesonephric tubule morphogenesis | 0.000606 | 0.033218 | 4 | Shared | 0.14 |
| BP | GO:0050650 | chondroitin sulfate proteoglycan biosynthetic process | 0.000692 | 0.03716 | 3 | Shared | 0.14 |
| BP | GO:0002062 | chondrocyte differentiation | 0.000708 | 0.037293 | 5 | Shared | 0.67 |
| BP | GO:0003338 | metanephros morphogenesis | 0.000849 | 0.043849 | 3 | Shared | 1.5 |
| KEGG | hsa00532 | Glycosaminoglycan biosynthesis - chondroitin sulfate / dermatan sulfate | 0.000208 | 0.030123 | 3 | Shared | 0.42 |

**Supplementary Table 5.** **Significantly enriched GO terms from the DMG of OA vs Control.** Data are sorted in rows based on the *q-value*. The KBD-associated DMGs were significantly enriched in skeletal system and limb associated pathways. We defined the fold change as the average PMD of DMGs involved in the pathway.

| GO Names | Ontology | Description | *p-value* | *q value* | #Genes | Shared | Fold change |
| --- | --- | --- | --- | --- | --- | --- | --- |
| BP | GO:0048562 | embryonic organ morphogenesis | 1.43E-09 | 9.54E-07 | 10 | Shared | 0.55 |
| BP | GO:0001501 | skeletal system development | 1.65E-09 | 9.54E-07 | 12 | Shared | 2.66 |
| BP | GO:0048568 | embryonic organ development | 3.99E-09 | 1.54E-06 | 11 | Shared | 0.79 |
| BP | GO:0048706 | embryonic skeletal system development | 2.41E-08 | 6.97E-06 | 7 | Shared | 1.49 |
| BP | GO:0048704 | embryonic skeletal system morphogenesis | 1.17E-07 | 2.70E-05 | 6 | Shared | 1.26 |
| BP | GO:0002062 | chondrocyte differentiation | 3.98E-07 | 7.02E-05 | 6 | Shared | 2.02 |
| BP | GO:0030326 | embryonic limb morphogenesis | 5.40E-07 | 7.02E-05 | 6 | Shared | 1.19 |
| BP | GO:0035113 | embryonic appendage morphogenesis | 5.40E-07 | 7.02E-05 | 6 | Shared | 1.19 |
| BP | GO:0051216 | cartilage development | 5.46E-07 | 7.02E-05 | 7 | Shared | 2.29 |
| BP | GO:0035115 | embryonic forelimb morphogenesis | 1.26E-06 | 0.000135 | 4 | Shared | 1.26 |
| BP | GO:0035107 | appendage morphogenesis | 1.40E-06 | 0.000135 | 6 | Shared | 1.19 |
| BP | GO:0035108 | limb morphogenesis | 1.40E-06 | 0.000135 | 6 | Shared | 1.19 |
| BP | GO:0048705 | skeletal system morphogenesis | 1.71E-06 | 0.000152 | 7 | Shared | 1.55 |
| BP | GO:0035136 | forelimb morphogenesis | 2.85E-06 | 0.000236 | 4 | Shared | 1.26 |
| BP | GO:0061448 | connective tissue development | 3.22E-06 | 0.000248 | 7 | Shared | 2.29 |
| BP | GO:0048736 | appendage development | 4.19E-06 | 0.000285 | 6 | Shared | 1.19 |
| BP | GO:0060173 | limb development | 4.19E-06 | 0.000285 | 6 | Shared | 1.19 |
| BP | GO:0002063 | chondrocyte development | 4.67E-06 | 0.0003 | 4 | OA-specific | 1.19 |
| BP | GO:0072283 | metanephric renal vesicle morphogenesis | 5.57E-06 | 0.000339 | 3 | Shared | 0.44 |
| MF | GO:0001228 | DNA-binding transcription activator activity, RNA polymerase II-specific | 8.60E-06 | 0.000497 | 8 | Shared | 0.50 |
| BP | GO:0007389 | pattern specification process | 9.06E-06 | 0.000499 | 8 | OA-specific | 1.12 |
| BP | GO:0072077 | renal vesicle morphogenesis | 1.03E-05 | 0.000544 | 3 | Shared | 0.44 |
| BP | GO:0060231 | mesenchymal to epithelial transition | 1.24E-05 | 0.000597 | 3 | Shared | -0.19 |
| BP | GO:0072087 | renal vesicle development | 1.24E-05 | 0.000597 | 3 | Shared | 0.44 |
| BP | GO:0060675 | ureteric bud morphogenesis | 1.74E-05 | 0.000804 | 4 | Shared | 0.18 |
| BP | GO:0072171 | mesonephric tubule morphogenesis | 1.85E-05 | 0.000814 | 4 | Shared | 0.18 |
| BP | GO:0003002 | regionalization | 1.90E-05 | 0.000814 | 7 | OA-specific | 0.73 |
| MF | GO:0001227 | DNA-binding transcription repressor activity, RNA polymerase II-specific | 2.32E-05 | 0.000957 | 6 | Shared | 0.52 |
| BP | GO:0072006 | nephron development | 2.50E-05 | 0.000995 | 5 | OA-specific | 0.41 |
| BP | GO:0072078 | nephron tubule morphogenesis | 3.00E-05 | 0.001134 | 4 | OA-specific | 0.18 |
| BP | GO:0072273 | metanephric nephron morphogenesis | 3.04E-05 | 0.001134 | 3 | Shared | 0.44 |
| BP | GO:0003281 | ventricular septum development | 3.18E-05 | 0.001149 | 4 | OA-specific | 0.02 |
| BP | GO:0072088 | nephron epithelium morphogenesis | 3.36E-05 | 0.001177 | 4 | OA-specific | 0.18 |
| BP | GO:0061333 | renal tubule morphogenesis | 3.74E-05 | 0.001237 | 4 | OA-specific | 0.73 |
| BP | GO:0072028 | nephron morphogenesis | 3.74E-05 | 0.001237 | 4 | OA-specific | 0.52 |
| BP | GO:0021675 | nerve development | 4.16E-05 | 0.001336 | 4 | OA-specific | 0.41 |
| BP | GO:0003205 | cardiac chamber development | 5.73E-05 | 0.001793 | 5 | Shared | 0.18 |
| BP | GO:0003338 | metanephros morphogenesis | 6.03E-05 | 0.001837 | 3 | Shared | 0.44 |
| BP | GO:0001656 | metanephros development | 7.07E-05 | 0.002071 | 4 | Shared | 0.02 |
| BP | GO:0048732 | gland development | 7.16E-05 | 0.002071 | 7 | OA-specific | 0.18 |
| BP | GO:0072080 | nephron tubule development | 7.39E-05 | 0.002085 | 4 | OA-specific | 0.18 |
| BP | GO:0060993 | kidney morphogenesis | 7.72E-05 | 0.002121 | 4 | OA-specific | 0.18 |
| BP | GO:0048566 | embryonic digestive tract development | 8.06E-05 | 0.002121 | 3 | OA-specific | -0.20 |
| BP | GO:0061326 | renal tubule development | 8.07E-05 | 0.002121 | 4 | OA-specific | 0.42 |
| BP | GO:0001657 | ureteric bud development | 9.17E-05 | 0.002353 | 4 | Shared | 0.44 |
| BP | GO:0072163 | mesonephric epithelium development | 9.56E-05 | 0.002353 | 4 | Shared | 0.18 |
| BP | GO:0072164 | mesonephric tubule development | 9.56E-05 | 0.002353 | 4 | Shared | -0.42 |
| BP | GO:0001708 | cell fate specification | 9.96E-05 | 0.0024 | 4 | Shared | 0.18 |
| BP | GO:0001823 | mesonephros development | 0.000112 | 0.002653 | 4 | Shared | 0.18 |
| BP | GO:0001763 | morphogenesis of a branching structure | 0.000118 | 0.002738 | 5 | OA-specific | 0.46 |
| BP | GO:0072210 | metanephric nephron development | 0.000124 | 0.002789 | 3 | OA-specific | 0.18 |
| BP | GO:0060562 | epithelial tube morphogenesis | 0.000125 | 0.002789 | 6 | OA-specific | 0.18 |
| BP | GO:0072009 | nephron epithelium development | 0.000141 | 0.003088 | 4 | Shared | 0.18 |
| BP | GO:0003279 | cardiac septum development | 0.000152 | 0.003262 | 4 | OA-specific | 0.18 |
| BP | GO:0035850 | epithelial cell differentiation involved in kidney development | 0.000155 | 0.003267 | 3 | Shared | 1.45 |
| BP | GO:0009952 | anterior/posterior pattern specification | 0.000169 | 0.003489 | 5 | OA-specific | 0.18 |
| BP | GO:0042471 | ear morphogenesis | 0.000188 | 0.003822 | 4 | OA-specific | 0.93 |
| BP | GO:0048701 | embryonic cranial skeleton morphogenesis | 0.000192 | 0.003829 | 3 | Shared | 0.44 |
| BP | GO:0048538 | thymus development | 0.000205 | 0.004026 | 3 | OA-specific | 0.68 |
| BP | GO:0032330 | regulation of chondrocyte differentiation | 0.000219 | 0.004227 | 3 | OA-specific | 0.18 |
| BP | GO:0021545 | cranial nerve development | 0.000265 | 0.00502 | 3 | OA-specific | 0.02 |
| BP | GO:0061005 | cell differentiation involved in kidney development | 0.000281 | 0.005105 | 3 | Shared | -0.19 |
| BP | GO:0048703 | embryonic viscerocranium morphogenesis | 0.000284 | 0.005105 | 2 | Shared | 0.79 |
| BP | GO:0003206 | cardiac chamber morphogenesis | 0.000287 | 0.005105 | 4 | OA-specific | -0.53 |
| BP | GO:0003231 | cardiac ventricle development | 0.000287 | 0.005105 | 4 | OA-specific | 0.95 |
| BP | GO:0060688 | regulation of morphogenesis of a branching structure | 0.000334 | 0.005857 | 3 | OA-specific | -0.80 |
| BP | GO:0003337 | mesenchymal to epithelial transition involved in metanephros morphogenesis | 0.000347 | 0.005989 | 2 | OA-specific | 1.23 |
| BP | GO:0072073 | kidney epithelium development | 0.000385 | 0.0065 | 4 | Shared | -0.3 |
| BP | GO:0007585 | respiratory gaseous exchange | 0.000393 | 0.0065 | 3 | OA-specific | -0.19 |
| BP | GO:0042733 | embryonic digit morphogenesis | 0.000393 | 0.0065 | 3 | OA-specific | 0.54 |
| BP | GO:0003161 | cardiac conduction system development | 0.000416 | 0.006771 | 2 | OA-specific | 0.77 |
| BP | GO:0003170 | heart valve development | 0.000436 | 0.00701 | 3 | OA-specific | 0.02 |
| BP | GO:0045165 | cell fate commitment | 0.000505 | 0.008007 | 5 | Shared | 1.04 |
| BP | GO:0001822 | kidney development | 0.000532 | 0.008322 | 5 | OA-specific | 0.06 |
| MF | GO:0015269 | calcium-activated potassium channel activity | 0.000571 | 0.008756 | 2 | OA-specific | 0.18 |
| BP | GO:0061035 | regulation of cartilage development | 0.000583 | 0.008756 | 3 | OA-specific | -0.33 |
| BP | GO:1904888 | cranial skeletal system development | 0.000583 | 0.008756 | 3 | Shared | 0.16 |
| BP | GO:0072001 | renal system development | 0.000684 | 0.010151 | 5 | OA-specific | 0.62 |
| BP | GO:1905331 | negative regulation of morphogenesis of an epithelium | 0.000751 | 0.010993 | 2 | OA-specific | 0.54 |
| BP | GO:0040037 | negative regulation of fibroblast growth factor receptor signaling pathway | 0.000954 | 0.013625 | 2 | OA-specific | 1.19 |
| BP | GO:0048557 | embryonic digestive tract morphogenesis | 0.000954 | 0.013625 | 2 | OA-specific | 0.41 |
| BP | GO:0061138 | morphogenesis of a branching epithelium | 0.001041 | 0.014684 | 4 | OA-specific | -0.04 |
| BP | GO:0001655 | urogenital system development | 0.001181 | 0.016461 | 5 | OA-specific | 1.23 |
| BP | GO:0009791 | post-embryonic development | 0.0012 | 0.016525 | 3 | Shared | 0.95 |
| BP | GO:2000738 | positive regulation of stem cell differentiation | 0.001303 | 0.017735 | 2 | OA-specific | 0.41 |
| BP | GO:0006029 | proteoglycan metabolic process | 0.001424 | 0.019149 | 3 | OA-specific | -0.03 |
| BP | GO:0043523 | regulation of neuron apoptotic process | 0.001463 | 0.019456 | 4 | OA-specific | -0.03 |
| BP | GO:0061217 | regulation of mesonephros development | 0.001565 | 0.020571 | 2 | OA-specific | 0.82 |
| CC | GO:0034705 | potassium channel complex | 0.001672 | 0.021666 | 3 | OA-specific | 0.54 |
| BP | GO:0030206 | chondroitin sulfate biosynthetic process | 0.001704 | 0.021666 | 2 | Shared | 0.41 |
| BP | GO:0072202 | cell differentiation involved in metanephros development | 0.001704 | 0.021666 | 2 | OA-specific | 0.44 |
| BP | GO:0045992 | negative regulation of embryonic development | 0.001849 | 0.02311 | 2 | OA-specific | 0.80 |
| BP | GO:0043583 | ear development | 0.001858 | 0.02311 | 4 | OA-specific | 0.79 |
| BP | GO:0030218 | erythrocyte differentiation | 0.002122 | 0.025724 | 3 | OA-specific | -0.22 |
| BP | GO:0003209 | cardiac atrium morphogenesis | 0.002157 | 0.025724 | 2 | OA-specific | 0.16 |
| MF | GO:0005227 | calcium activated cation channel activity | 0.002157 | 0.025724 | 2 | OA-specific | -0.24 |
| BP | GO:0021602 | cranial nerve morphogenesis | 0.002157 | 0.025724 | 2 | OA-specific | 0.56 |
| BP | GO:0050650 | chondroitin sulfate proteoglycan biosynthetic process | 0.002319 | 0.027376 | 2 | Shared | 0.06 |
| BP | GO:0051402 | neuron apoptotic process | 0.002399 | 0.02803 | 4 | OA-specific | -0.03 |
| BP | GO:0021772 | olfactory bulb development | 0.002487 | 0.028769 | 2 | OA-specific | -0.54 |
| BP | GO:0034101 | erythrocyte homeostasis | 0.002573 | 0.02947 | 3 | OA-specific | -0.19 |
| BP | GO:0021988 | olfactory lobe development | 0.00266 | 0.02959 | 2 | OA-specific | 0.79 |
| BP | GO:0045880 | positive regulation of smoothened signaling pathway | 0.00266 | 0.02959 | 2 | OA-specific | -0.04 |
| CC | GO:0098686 | hippocampal mossy fiber to CA3 synapse | 0.00266 | 0.02959 | 2 | OA-specific | -0.19 |
| BP | GO:0003014 | renal system process | 0.002783 | 0.03066 | 3 | OA-specific | -0.10 |
| BP | GO:0009954 | proximal/distal pattern formation | 0.002839 | 0.030868 | 2 | OA-specific | 0.69 |
| BP | GO:2000736 | regulation of stem cell differentiation | 0.002855 | 0.030868 | 3 | OA-specific | -0.44 |
| BP | GO:0003230 | cardiac atrium development | 0.003023 | 0.031681 | 2 | OA-specific | -0.35 |
| BP | GO:0019228 | neuronal action potential | 0.003023 | 0.031681 | 2 | OA-specific | 0.53 |
| BP | GO:0040036 | regulation of fibroblast growth factor receptor signaling pathway | 0.003023 | 0.031681 | 2 | OA-specific | 0.55 |
| BP | GO:0007611 | learning or memory | 0.00304 | 0.031681 | 4 | OA-specific | 0.79 |
| BP | GO:0048608 | reproductive structure development | 0.003114 | 0.032168 | 5 | OA-specific | 0.11 |
| BP | GO:0007548 | sex differentiation | 0.003181 | 0.0322 | 4 | OA-specific | -0.03 |
| BP | GO:0110111 | negative regulation of animal organ morphogenesis | 0.003213 | 0.0322 | 2 | OA-specific | 0.47 |
| BP | GO:0061458 | reproductive system development | 0.003218 | 0.0322 | 5 | OA-specific | -0.57 |
| BP | GO:2000027 | regulation of animal organ morphogenesis | 0.003229 | 0.0322 | 4 | OA-specific | -0.79 |
| MF | GO:0005267 | potassium channel activity | 0.003313 | 0.032479 | 3 | OA-specific | -0.03 |
| CC | GO:0005795 | Golgi stack | 0.003313 | 0.032479 | 3 | OA-specific | -0.57 |
| BP | GO:0090596 | sensory organ morphogenesis | 0.003476 | 0.033792 | 4 | OA-specific | 0.78 |
| BP | GO:0030204 | chondroitin sulfate metabolic process | 0.003609 | 0.034795 | 2 | OA-specific | -0.24 |
| BP | GO:0003007 | heart morphogenesis | 0.003683 | 0.035212 | 4 | OA-specific | 0.30 |
| BP | GO:0003156 | regulation of animal organ formation | 0.003815 | 0.035885 | 2 | OA-specific | -0.54 |
| CC | GO:0016235 | aggresome | 0.003815 | 0.035885 | 2 | OA-specific | 0.56 |
| BP | GO:0035270 | endocrine system development | 0.00399 | 0.037224 | 3 | OA-specific | 0.77 |
| BP | GO:0055007 | cardiac muscle cell differentiation | 0.00408 | 0.03776 | 3 | OA-specific | 0.61 |
| BP | GO:0048565 | digestive tract development | 0.004171 | 0.038299 | 3 | OA-specific | -0.09 |
| KEGG | hsa00532 | Glycosaminoglycan biosynthesis - chondroitin sulfate / dermatan sulfate | 0.000970 | 0.034729 | 2 | Shared | 0.46 |
